# The gut microbiome from patients with schizophrenia modulates the glutamate-glutamine-GABA cycle and schizophrenia-relevant behaviors in mice

**DOI:** 10.1126/sciadv.aau8317

**Published:** 2019-02-06

**Authors:** Peng Zheng, Benhua Zeng, Meiling Liu, Jianjun Chen, Junxi Pan, Yu Han, Yiyun Liu, Ke Cheng, Chanjuan Zhou, Haiyang Wang, Xinyu Zhou, Siwen Gui, Seth W. Perry, Ma-Li Wong, Julio Licinio, Hong Wei, Peng Xie

**Affiliations:** 1 Department of Neurology, The First Affiliated Hospital of Chongqing Medical University, Chongqing, China.; 2 Institute of Neuroscience and the Collaborative Innovation Center for Brain Science, Chongqing Medical University, Chongqing, China.; 3 Chongqing Key Laboratory of Neurobiology, Chongqing, China.; 4 Department of Laboratory Animal Science, College of Basic Medical Sciences, Third Military Medical University, Chongqing, China.; 5 Department of Gastroenterology, The First Affiliated Hospital of Chongqing Medical University, Chongqing, China.; 6 College of Life Sciences, Chongqing Medical University, Chongqing, China.; 7 The M.O.E. Key Laboratory of Laboratory Medical Diagnostics, the College of Laboratory Medicine, Chongqing Medical University, Chongqing, 400016, China.; 8 Department of Psychiatry, College of Medicine, SUNY Upstate Medical University, Syracuse, NY, USA.; 9 Precision Medicine Institute, The First Affiliated Hospital, Sun Yat-sen University, Guangzhou, Guangdong 510080, China.

## Abstract

Schizophrenia (SCZ) is a devastating mental disorder with poorly defined underlying molecular mechanisms. The gut microbiome can modulate brain function and behaviors through the microbiota-gut-brain axis. Here, we found that unmedicated and medicated patients with SCZ had a decreased microbiome α-diversity index and marked disturbances of gut microbial composition versus healthy controls (HCs). Several unique bacterial taxa (e.g., Veillonellaceae and Lachnospiraceae) were associated with SCZ severity. A specific microbial panel (Aerococcaceae, Bifidobacteriaceae, Brucellaceae, Pasteurellaceae, and Rikenellaceae) enabled discriminating patients with SCZ from HCs with 0.769 area under the curve. Compared to HCs, germ-free mice receiving SCZ microbiome fecal transplants had lower glutamate and higher glutamine and GABA in the hippocampus and displayed SCZ-relevant behaviors similar to other mouse models of SCZ involving glutamatergic hypofunction. Together, our findings suggest that the SCZ microbiome itself can alter neurochemistry and neurologic function in ways that may be relevant to SCZ pathology.

## Teaser

New findings suggest that schizophrenia may be linked to gut bacteria.

## INTRODUCTION

Schizophrenia (SCZ) is a devastating illness affecting approximately 0.5 to 1% of the general population worldwide (*1*). Previously, researchers have focused on analysis of the human genome to determine the pathogenesis of SCZ (*2*). Genome-wide association (GWAS) analysis of 36,000 patients identified 108 susceptibility loci for SCZ (*3*). However, the identified associations likely account for only about 4% of the variance in SCZ. Thus, we should also seek to identify the role of non-human genetic factors in the onset of SCZ.

The gastrointestinal (GI) tract is a complex ecosystem containing a large number of resident microorganisms (*4*). Recent evidence suggests that the gut microbiota could modulate brain function and behaviors via the “microbiota-gut-brain” (MGB) axis (*5*, *6*). For example, gut microbiota have been reported to be associated with alterations in anxiety (*7*), memory (*8*), cognition (*9*), and locomotor activity (*10*). Our groups recently showed that modulation of gut microbiota using the germ-free (GF) method or antibiotics could result in depressive-like behaviors (*11*, *12*). These findings highlight the novel possibility that disturbances of gut microbiota or the MGB axis may contribute to the onset of psychiatric disorders.

The relationships between the MGB axis and the SCZ are not yet fully understood. Emerging clinical and preclinical studies indicate potential associations between a disturbed gut microbiome and SCZ (*13*). Epidemiological studies have shown that prenatal microbial infection resulted in a 10- to 20-fold increased risk of developing SCZ (*14*). In addition, SCZ is frequently comorbid with GI disorders that are characterized by alterations of gut microbial communities (*15*). In animal studies, gut microbiota are crucial in postnatal development and maturation of neural, immune, and endocrine systems (*16*), and these behavioral and physiological processes are frequently impaired in patients with SCZ (*17*). These aforementioned studies suggest that disturbance of the MGB axis may be associated with development of SCZ.

To address this issue, a culture-independent, 16*S* ribosomal RNA (16*S* rRNA) gene sequence–based approach was used to compare the gut microbial communities of patients with SCZ and healthy controls (HCs) to evaluate whether microbiotal dysbiosis was linked with schizophrenic episodes or the severity of schizophrenic symptoms. Then, we transferred gut microbiota from patients with SCZ into GF mice to test whether SCZ-relevant behavioral phenotypes were transmissible via their gut microbiome. Last, to capture functional readout of microbial activity, we performed comparative metagenomic and metabolomic analyses of samples from the mice harboring “SCZ microbiota” versus “HC microbiota” to determine the potential mechanistic pathways by which the disturbed gut microbiota may modulate host physiology and behavior.

## RESULTS

### Human studies

#### Clinical characteristics of recruited participants

A total of 63 patients with SCZ and 69 HCs were recruited for this study. There were no significant differences in age, gender, or body mass index between the two groups. All patients with SCZ were undergoing some symptoms of this illness. The Positive and Negative Syndrome Scale Score (PANSS) measured the severity of schizophrenic symptoms and ranged from 45 to 120. Detailed characteristics of the recruited participants are shown in table S1 and “study design” under Materials and Methods.

#### Lower within-sample microbial diversity in patients with SCZ

**Fig. 1:**
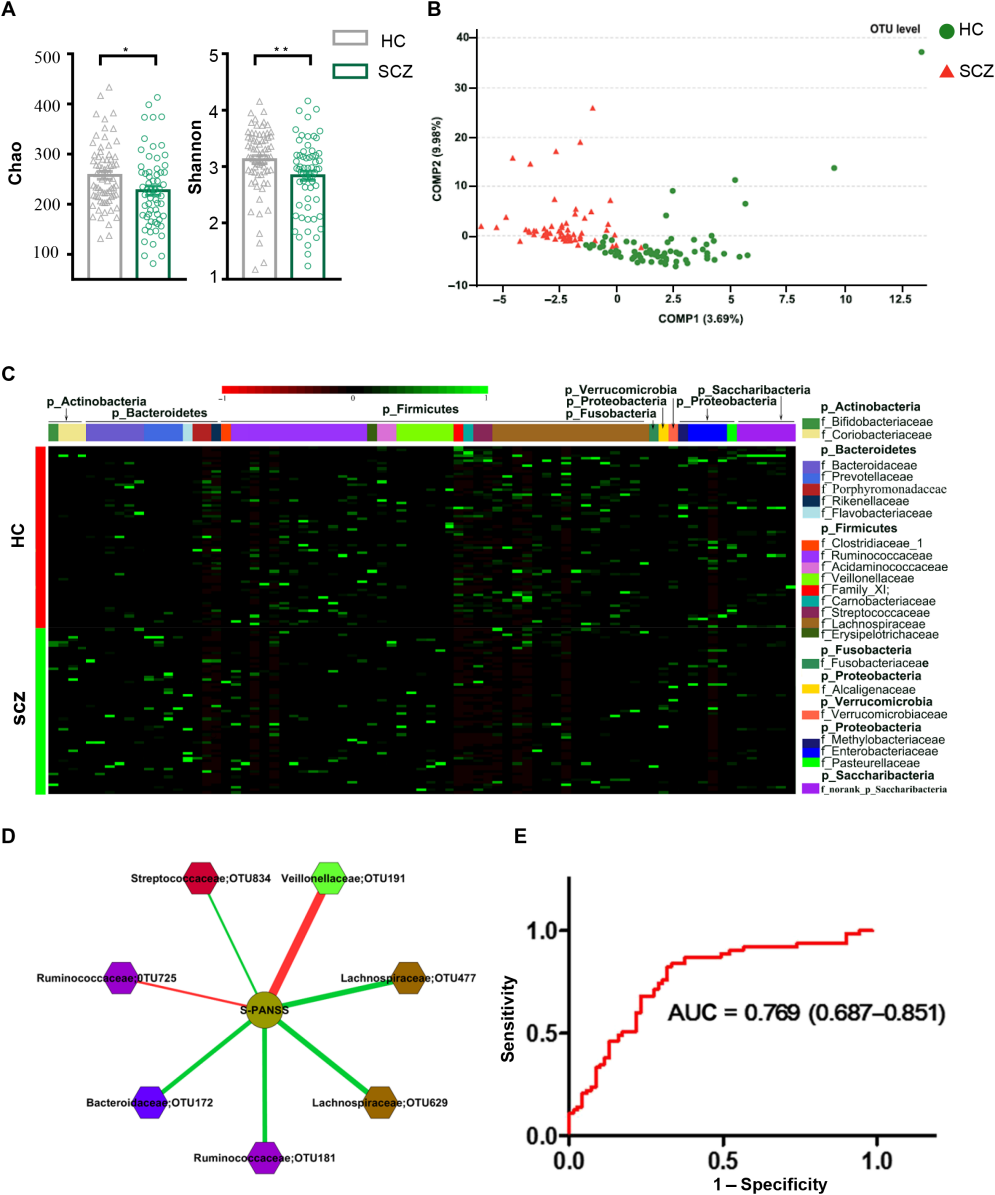
Gut microbial characteristics of SCZ. (**A**) α-Phylogenetic diversity analysis showed that patients with SCZ were characterized by lower microbial richness (Chao, *P < 0.05) and diversity (Shannon, **P < 0.01) indices relative to HCs. (**B**) At the OTU level, partial least-squares discriminant analysis (PLS-DA) showed that gut microbiota composition of patients with SCZ was greatly different from that of HCs. (**C**) Heat map of the 77 discriminative OTU abundances between patients with SCZ and HCs; 23 up-regulated OTUs in SCZ are arranged on the left part of the image, and 54 decreased OTUs are arranged on the right part. The taxonomic assignment of each OTU is provided on the right column. (**D**) OTUs related to SCZ symptom severity (quantitation with PANSS). The red line designates negative correlation between PANSS and microbes, while the green line shows positive correlation. Lachnospiraceae and Ruminococcaceae are shown twice because they both had two different OTUs correlated with PANSS (see Results). (**E**) ROC analysis showed that the combination of Aerococcaceae, Bifidobacteriaceae, Brucellaceae, Pasteurellaceae, and Rikenellaceae can distinguish patients with SCZ from HCs with an AUC of 0.769.

In total, we obtained 2,905,956 high-quality reads across all samples with an average length of 439.18. These reads were clustered into 864 OTUs (operational taxonomic units) at 97% sequence similarity. A Venn diagram showed that 744 of 864 OTUs were detected in the two groups, while 56 and 64 OTUs were unique to patients with SCZ and HCs subjects, respectively (fig. S1A). Most rarefaction curves tended to approach the saturation plateau, suggesting that the sequencing depth was enough to cover the whole bacterial diversity (fig. S1B). Within-sample α-phylogenetic diversity analysis showed that both microbial richness indices (Chao) and diversity indices (Shannon) were lower in patients with SCZ when compared to those of HCs (Fig. 1A). These findings suggest that the microbial compositions of patients with SCZ were characterized by lower within-sample diversity.

#### Patients with SCZ exhibit altered gut microbiota

To determine whether the microbial composition of patients with SCZ was substantially different from that of HC subjects, we carried out β-diversity analysis and found obvious differences in gut microbial composition between the two groups from order to OTU levels (fig. S1, C to F and Fig. 1B). At the phylum or class level, SCZ and HC subjects showed a separation trend (fig. S1, G and H). We also performed analyses to determine whether the global microbial phenotypes were substantially affected by potential confounding variables (i.e., sex or antipsychotic drugs). The patients with SCZ or HCs were not clustered on the basis of these variables, suggesting that global microbial phenotypes were not greatly influenced by sex or medication status (fig. S2, A to C).

To further identify the gut microbiota responsible for discriminating patients with SCZ from HC subjects, we carried out Linear discriminant analysis Effect Size (LEfSe). This analysis identified 77 differential OTUs responsible for the discrimination between the two groups (Fig. 1C and table S2A). Twenty-three of 77 OTUs were increased in patients with SCZ compared to HC subjects, and those OTUs mainly belonged to the bacterial taxonomic families Veillonellaceae (five OTUs), Prevotellaceae (four OTUs), Bacteroidaceae (three OTUs), and Coriobacteriaceae (two OTUs). The remaining 54 OTUs were decreased in patients with SCZ relative to HC subjects, and they belonged to the bacterial families Lachnospiraceae (16 OTUs), Ruminococcaceae (12 OTUs), Norank (5 OTUs), and Enterobacteriaceae (4 OTUs).

#### Dysbiosis of gut microbiota in SCZ is specific relative to that of major depressive disorder

To determine whether these altered OTUs were relatively specific to SCZ versus other neuropsychiatric disorders, we compared key differential bacterial taxa observed in SCZ and major depression. Previously, we identified 54 OTUs able to discriminate between depressed and HC subjects (*12*). Compared to the HC group, only 15.3% OTUs (8 of 52) belonging to Ruminococcaceae were up-regulated, and 30.3% OTUs (24 of 79) belonging to Acidaminococcaceae, Bacteroidaceae, Ruminococcaceae, and Veillonellaceae were synchronously down-regulated, in both SCZ and depression groups (fig. S3). These findings indicate that the altered gut microbial composition observed in SCZ is specific relative to the gut microbiome changes we observed in depression.

#### Microbial markers for symptomatic severity and diagnosis in SCZ

To identify the OTUs related to severity of schizophrenic symptoms, we performed correlation analysis. Veillonellaceae OTU191 was negatively correlated with PANSS, whereas Bacteroidaceae OTU172, Streptococcaceae OTU834, and two Lachnospiraceae OTUs (477 and 629) were positively correlated with PANSS. Ruminococcaceae also had two OTUs correlated with PANSS, one negatively correlated (OTU725) and the other positively correlated (OTU181) (Fig. 1D).

To identify key discriminative microbial markers, we performed a stepwise regression analysis based on relative abundance of different gut microbes. This analysis showed that the most significant deviations between SCZ and HC subjects occurred for the bacterial families Aerococcaceae, Bifidobacteriaceae, Brucellaceae, Pasteurellaceae, and Rikenellaceae. An ROC (receiver operating characteristic) analysis showed that this microbial panel enabled discrimination of patients with SCZ from HC subjects with an area under the curve (AUC) of 0.769 (Fig. 1E), suggesting potential diagnostic value for SCZ.

### Animal studies

#### Gut microbiome transplantation from patients with SCZ induces SCZ-relevant behaviors in GF recipient mice

**Fig. 2:**
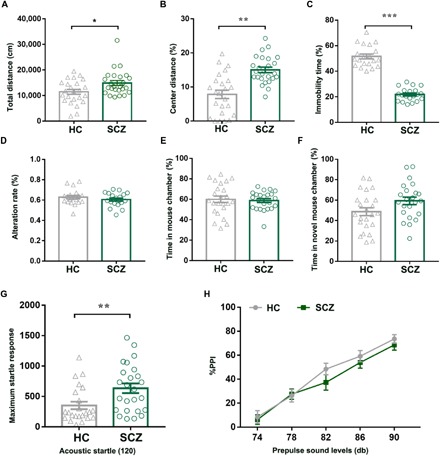
Behavioral comparisons between the SCZ microbiota recipient mice and the HC microbiota recipient mice. (**A** and **B**) Open-field test. Compared to the HC microbiota recipient mice, the total distance (A) and proportion (B) of central distance traveled in 30 min were significantly increased in the SCZ microbiota recipient mice (HC, *n* = 24; SCZ, *n* = 25). (**C**) Forced swimming test. Compared to the HC microbiota recipient mice, the duration of immobility was significantly decreased in the SCZ microbiota recipient mice (*n* = 20 per group). (**D**) Y-maze test. There was no difference in the alteration rate between the two groups (*n* = 20 per group). (**E** and **F**) Sociability and social novelty preference test. In the sociability test (E) and social novelty preference tests (F), the time investigating the chamber containing a novel mouse versus the time investigating both chambers was indistinguishable between the two groups (HC, *n* = 24; SCZ, *n* = 23). (**G** and **H**) Prepulse inhibition (PPI) test. (G) The SCZ microbiota recipient mice displayed an exaggerated startle response to high-decibel tones (120 db) relative to the HC microbiota recipient mice. (H) Increasing prepulse intensity led to increased PPI magnitude in both microbiota recipient groups; however, the PPI magnitude did not differ between the two groups. All data were presented as means ± SEM. *P < 0.05, **P < 0.01, ***P < 0.001 using nonparametric tests).

To determine whether SCZ-relevant behavioral phenotypes might be linked with disturbed gut microbiota, we performed fecal microbiota transplantation (FMT) experiments. The global gut microbial phenotypes of the randomly selected subset samples used for these FMT experiments were representative of their full population distributions (fig. S4A). In the open-field test, the SCZ microbiota recipient mice showed hyperactivity (greater total distance traveled; Fig. 2A) and reduced anxiety (more travel in the exposed center region away from the walls; Fig. 2B). Similarly, the duration of immobility in the forced swimming test was significantly decreased in the SCZ microbiota recipient mice compared to the HC microbiota recipient mice (Fig. 2C), suggesting decreased depressive-like (and more active) behavior. Cognitive behaviors were also measured using Y-maze, sociability, and social novelty preference tests, as well as the prepulse inhibition (PPI) test. In the Y-maze test, there was no difference between the two groups (Fig. 2D). In the sociability test, the time investigating the chamber containing a mouse versus the alternative empty chamber did not differ between groups (Fig. 2E). Furthermore, the time investigating a novel versus a familiar mouse was also statistically indistinguishable (*P* = 0.100) between the groups in the social novelty preference test (Fig. 2F). Compared to the HC microbiota recipient mice, the SCZ microbiota recipient mice displayed an exaggerated startle response to high-decibel tones (120 db) (Fig. 2G), but PPI did not differ between the two groups (Fig. 2H). Collectively, these behavioral tests showed that mice transplanted with SCZ microbiota displayed locomotor hyperactivity, decreased anxiety- and depressive-like behaviors, and increased startle responses, suggesting that the disturbed microbial composition of SCZ microbiota recipient mice was associated with several endophenotypes characteristic of mouse models of SCZ (see Discussion).

#### The key discriminative microbial markers found in patients with SCZ were successfully colonized in the SCZ microbiota recipient mice

To determine whether the discriminative microbial markers characteristic of SCZ were successfully colonized in the SCZ microbiota recipient mice, we characterized gut microbial compositions. Overall, the microbial phenotypes of the SCZ microbiota recipient mice were greatly different from those of the HC microbiota recipient mice (fig. S4B). Stepwise regression analysis showed that the most significant discrimination between these two groups was attributable to the bacterial families Aerococcaceae and Rikenellaceae, and their combined microbial markers could completely discriminate the SCZ microbiota recipient mice from their HC counterparts with an AUC of 1, which represents 100% discrimination accuracy (fig. S4C). Furthermore, identical changes in Aerococcaceae and Rikenellaceae composition were seen in both patients with SCZ and the SCZ microbiota recipient mice (fig. S4, D and E).

#### Perturbed gut-brain amino acid and lipid metabolism in SCZ microbiota recipient mice

**Fig. 3:**
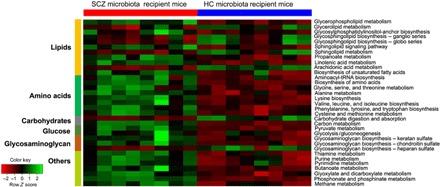
Metagenomic analysis identified differential KEGG pathways between SCZ microbiota and HC microbiota recipient mice. The altered differential KEGG Orthologs (KOs) were mainly involved in 33 disturbed metabolic pathways. Eight of 33 pathways were up-regulated, and the remaining pathways were down-regulated in the SCZ microbiota recipient mice compared to HC microbiota recipient mice (*n* = 8 per group). tRNA, transfer RNA.

To characterize the functions encoded by the gut microbiotal DNA, we performed whole-genome shotgun sequencing of cecum stool samples obtained from the SCZ microbiota and the HC microbiota recipient mice at week 2 after FMT. Pathway-enrichment analysis revealed that eight pathways were increased, whereas 25 pathways were decreased in the SCZ microbiota recipient mice compared to the HC microbiota recipient mice (Fig. 3 and table S2B), and SCZ microbiota recipient mice were characterized by enrichment of genes for lipid and amino acid metabolism.

**Fig. 4:**
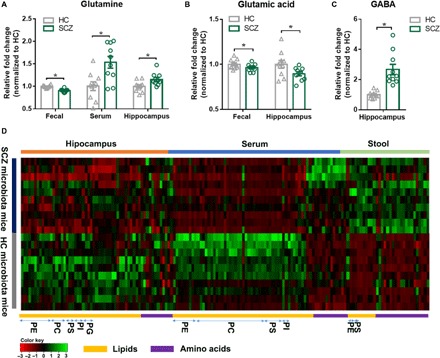
Altered metabolites in stool, serum, and hippocampus. (**A** to **C**) Key metabolites glutamine (A), glutamate (B), and GABA (C) related to glutamatergic neurotransmission metabolism were significantly changed in the SCZ microbiota mice (*n* = 10 per group). All data were presented as means ± SEM. *P < 0.05 using Student’s *t* test. (**D**) A heat map shows the altered metabolites in stool, serum, and hippocampus. Functional clustering analysis showed that these differentially expressed fecal, serum, and hippocampal metabolites were consistently involved in amino acid and lipid metabolism (*n* = 10 per group).

We further performed nontargeted metabolomics to determine whether or which metabolisms modulated by the gut microbiome were paralleled by an altered MGB axis. We found that the metabolomic profiles of fecal, serum, and hippocampal samples obtained from the SCZ microbiota recipient mice were substantially different from those of the HC microbiota recipient mice (fig. S5, A to G). We identified the differentially expressed metabolites between the two groups (table S3, A to C). Of particular relevance to SCZ, we measured differences in central nervous system and peripheral glutamate-glutamine–GABA (γ-aminobutyric acid) metabolism, and in the SCZ microbiota compared to the HC microbiota recipient mouse samples, we found elevated glutamine in the serum and hippocampus, decreased glutamate (glutamic acid) in the stool and hippocampus, and increased GABA in the hippocampus (Fig. 4, A to C and fig. S6). Serum glutamate and GABA, as well as fecal GABA, were not different between the two groups (fig. S7, A and B). Significant differences between HC and SCZ subjects were seen in these three metabolites in cortex, but not in cerebellum or striatum (fig. S7, C to E), thus confirming that the observed metabolic disturbances are localized to the glutamate-rich brain regions (i.e., hippocampus and cortex) that are most consistently implicated in glutamate-glutamine-GABA disruptions in SCZ (*18*–*20*) and that are relevant to the behavioral endophenotypes observed (*21*–*24*). Functional clustering analysis showed that these differentially expressed fecal, serum, and hippocampal metabolites were consistently involved in amino acid metabolism, for example, in the glutamate-glutamine-GABA cycle (Fig. 4D and fig. S6), transport of several amino acids (fig. S6), and lipid metabolism (e.g., glycerophospholipids; Fig. 4D). Overall, the results for the SCZ microbiota mice suggested alterations in the glutamate-glutamine-GABA cycle and amino acid metabolism and transport. The altered lipids were mainly glycerophospholipids including phosphatidylethanolamines (PEs), phosphoserines (PSs), phosphatidylcholines (PCs), or phosphatidylinositol (PIs) and were generally decreased in the serum and hippocampus of SCZ recipient mice (Fig. 4D). Together, these metagenomic and metabolomic findings suggest that alterations in gut microbiota may be associated with SCZ pathophysiology through MGB amino acid and lipid metabolic pathways.

## DISCUSSION

**Fig. 5:**
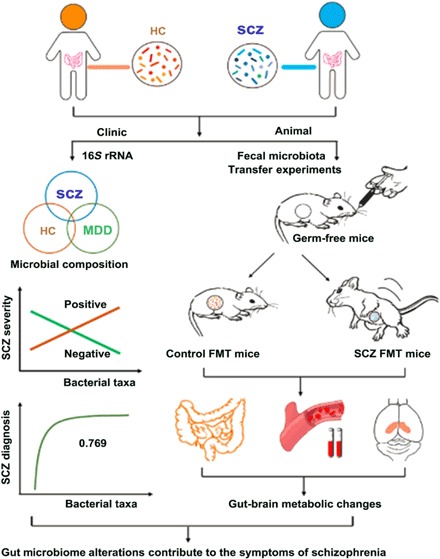
The workflow diagram for this study. SCZ is associated with dysbiosis of gut microbial composition, which is distinct from that seen in major depressive disorder (MDD). This alteration can result in host gut-brain axis metabolic and neurobehavioral changes relevant to SCZ.

Gut microbiota can influence brain function and behaviors through the MGB axis and thus may predispose the onset of various neuropsychiatric disorders (*25*). Here, we found profound gut microbiota alterations in patients with SCZ relative to HC subjects. We identified unique bacterial taxa that were strongly associated with SCZ severity. Moreover, a specific microbial panel enabled discrimination of SCZ from HC subjects with an AUC of 0.769. Our animal experiments of GF mice colonization with human SCZ microbiota resulted in SCZ-relevant behavioral changes similar to those observed in glutamatergic mouse models of SCZ (see Fig. 5 for study flow). These behavioral phenotypes were not seen in the control mice colonized with human HC microbiota. Consistent with these behavioral changes, the mice receiving gut microbiome transfers from patients with SCZ displayed disturbances of microbial genes and host metabolites involved in amino acid and lipid metabolism, including glutamate, which has been strongly implicated in SCZ pathology. Our findings support the possibility that alterations of gut microbiota may potentially participate in the onset and/or pathology of SCZ through modulating MGB metabolic pathways.

We also found that the microbial composition of patients with SCZ was less diverse, i.e., was associated with lower α-diversity scores, than that of HC individuals. Generally speaking, a high α-diversity is thought to represent a marker of “good” health status. Here, the lower α-diversity suggests an overall abnormal microbial status within patients with SCZ. Previously, we found that there was no difference in gut microbiome α-diversity between depressed individuals and HC subjects (*12*). Moreover, the gut microbiome changes observed in patients with SCZ herein are greatly different from those seen in depressed individuals. These findings suggest that dysbiosis of gut microbiota in SCZ is distinct from the microbiota characteristic of depression. Here, patients with SCZ were characterized mainly by decreased relative abundance of Lachnospiraceae (16 OTUs) and Ruminococcaceae (12 OTUs). The Lachnospiraceae and Ruminococcaceae are two of the most abundant families from the order Clostridiales observed in the gut microbiome and have been linked with the maintenance of gut health. The decrease in these two gut microbiota species suggests abnormal microbial status in SCZ, and follow-up studies using microbial metagenomics and culturomics may further define how these and other specific gut microbiota species may affect SCZ etiopathogenesis. In the context of these findings, several events shown to influence composition of the gut microbiome, especially during the microbiome’s establishment/dynamic period in infancy—e.g., cesarean versus vaginal birth, breast versus formula feeding, or early life antibiotic treatment—have all been associated, to some degree, with risk or onset of SCZ (*26*). While these links are not definitive, they provide compelling avenues for further investigation. In these contexts, it is unlikely a coincidence that the antibiotic minocycline has shown significant potential as an adjunctive therapy for SCZ (*27*). Together, our findings have potential clinical diagnostic value, with treatment implications, and lay the groundwork for further identification of “signature patterns” of defined gut microbes in SCZ.

We also demonstrated that transfer of human SCZ microbiomes to mice induced changes in gut microbial composition and SCZ-relevant behavioral phenotypes, compared to mice receiving HC microbiomes. Behavioral phenotypes seen in mouse models relevant to SCZ can be somewhat nonspecific and have relevance to multiple human psychiatric disorders, can vary substantially by manner of induction, and can have variable refractoriness to antipsychotics typically used to treat SCZ (*21*). These represent just some of the difficulties in establishing uniform and consistent mouse models with high predictive validity for SCZ and other psychiatric disease. However, one of the key findings here is that mice receiving SCZ microbiome were hyperactive relative to mice receiving HC microbiome (Fig. 2, A to C), and hyperactivity in these and similar behavioral tests is one of the most consistent findings across multiple rodent models of SCZ (*21*) and hypoglutamatergic rodent models of SCZ in particular (*23*, *24*). We also observed a significant increase in startle response (Fig. 2G), which is characteristic of the SCZ dysbindin knockout model that involves hypoactive glutamatergic function (*22*), as does our SCZ microbiome model herein. In addition, some behavioral tests including social interactions and PPI of acoustic startle were not significantly different between SCZ and HC microbiome mice, suggesting that alterations of gut microbiome are associated with some (but not all) endophenotypes characteristic of mouse models of SCZ (*21*). Together, these results suggest that our SCZ microbiome–induced model shares many features with other well-characterized SCZ rodent models that involve decreased glutamate signaling. These behavioral results, together with our metabolomic and metagenomic data, strongly suggest that the SCZ microbiome transplantation affects the same SCZ-relevant behavioral pathways that are affected in other SCZ rodent models, particularly glutamate circuits.

Consistent with these behavioral data characteristic of other hypoglutamatergic rodent models of SCZ, we identified decreased brain glutamate and disruptions in the glutamate-glutamine-GABA cycle and altered amino acid and lipid metabolism as possible mechanistic targets that may underlie the altered behavior in the SCZ microbiota recipient mice. Glutamatergic neurotransmission (*18*) and, in particular, decreased hippocampal glutamate (*19*, *20*) have been widely implicated in the pathophysiology of SCZ. In the SCZ microbiota recipient mice, the observed decreased hippocampal glutamate levels, together with higher glutamine and GABA, may reflect reduced glutamatergic neurotransmission, increased production of GABA from glutamate or decreased breakdown of GABA into glutamate, increased conversion of glutamate to glutamine (via glutamine synthetase) or decreased conversion of glutamine to glutamate (via glutaminase), and/or altered transport or reuptake of any of the above. Future experiments will seek to clarify these observed alterations in glutamate-glutamine-GABA metabolism. Within the brain, the localization and confinement of our observed metabolic disturbances to hippocampus and cortex (Fig. 4, A to C, and fig. S7, C to E) align with evidence that these are the brain regions most consistently implicated in glutamate-glutamine-GABA disruptions in SCZ (*18*–*20*) and affected in the endophenotypes observed herein (*21*–*24*) and suggest a targeted pathogenic functional specificity resulting from the SCZ microbiota transplantation. However, future studies characterizing metabolic disturbances in other glutamate-glutamine-GABA–rich brain regions with significant relevance to SCZ, such as the amygdala, are also needed.

We also found significant changes in MGB axis lipid species, especially involving glycerophospholipid metabolism, in the SCZ microbiota versus HC microbiota mice. Consistent with these findings, significant disturbances in serum and brain lipids have been observed in patients with SCZ (*28*). The glycerophospholipids including PEs, PSs, PCs, and PIs are major components of myelin and neuronal membranes and are key regulators of synaptic function. Our results showed that these glycerophospholipids and their related metabolites were typically decreased in the hippocampus and serum, yet increased in fecal samples, of SCZ versus HC microbiota mice, respectively. Whether this observed increase in waste (i.e., fecal) glycerophospholipid metabolites was causal or consequent to, or independent of, the corresponding decreases in serum and hippocampal metabolites requires further investigation. Regardless, these findings suggest that the SCZ gut microbiome resulted in lower glycerophospholipid content in the periphery and brains of SCZ microbiota recipient mice, pathologies that are consistent with the synaptic deficits and disconnectivity that are thought to underlie SCZ (*29*).

There were some possible confounds common to many clinical and translational SCZ studies that warrant mention. First, most of the patients with SCZ were taking antipsychotic medication. Recruitment of unmedicated patients with SCZ is very difficult, because once initial SCZ-like symptoms of hallucination, delusion, or impulsive behavior appear, pharmacotherapy is initiated before diagnosis and continues after formal SCZ diagnosis. However, to mitigate this concern relevant to our experiments, we found that the distributions of global microbial phenotypes did not vary between medicated and unmedicated patients with SCZ (fig. S2, B and C), or with respect to medication type (fig. S2C), indicating that antipsychotic treatment was not a prominent confounding variable that was likely to affect the interpretation of our results. Second, all recruited subjects were symptomatic for SCZ, and this could be why the distribution of global microbial phenotypes did not vary between medicated and unmedicated patients with SCZ. Studies with larger numbers of unmedicated patients are required to validate and extend the current findings. Nonetheless, as yet, we cannot entirely rule out the possibility that the endophenotypic effects observed in the SCZ microbiota mice were a function of the medicated microbiome rather than the SCZ disease state per se.

Together, we provide seminal evidence that SCZ is associated with changes in gut microbiota composition that are both specific to SCZ and correlated with symptom severity. Moreover, we found that changes in the gut microbiota resulting from human SCZ fecal microbiome transfer to mice lead to hypoglutamatergia and onset of SCZ-relevant behaviors characteristic of SCZ rodent models, accompanied by gut-brain axis metabolic changes. Our findings provide a novel framework for understanding the mechanisms of SCZ through the MGB axis and may lead to new diagnostic and treatment strategies.

## MATERIALS AND METHODS

### Study design

The study protocol was approved by the Ethics Committee of Chongqing Medical University. All participants signed a written informed consent before any procedure was carried out. In total, 63 SCZ and 69 HC subjects were recruited from the First Affiliated Hospital of Chongqing Medical University. Here, most of the patients with SCZ were treated with a single antipsychotic drug including clozapine (*n* = 15), risperidone (*n* = 14), olanzapine (*n* = 9), chlorpromazine (*n* = 5), aripiprazole (*n* = 3), and quetiapine (*n* = 3), while the remaining patients were treated with two of the above drugs in combination (*n* = 9) or were unmedicated (*n* = 5). In clinical practice, the diversity of SCZ drugs is determined by the diversity of clinical symptoms and the heterogeneity of pathological mechanisms. SCZ was diagnosed by Structured Psychiatric Interview using Diagnostic and Statistical Manual of Mental Disorders, 4th Edition (DSM-IV) criteria by two senior psychiatrists (*30*). All participants did not have any physical or other mental disorders or illicit drug use, and they also had not taken any antibiotics, probiotics, or prebiotics within 1 month before sampling. Fresh stool samples were collected from each participant and immediately frozen at −80°C until analysis. Sample sizes were determined empirically on the basis of previous studies, are similar or larger than those in related published literature, and are as stated here or in each of the figure legends. To accurately reflect the clinical reality, we did not exclude outliers. Statistical analyses were performed as described below.

### DNA extraction, polymerase chain reaction amplification, and Illumina MiSeq sequencing

The Illumina MiSeq sequencing protocol was based on our previous published literature (*12*). Briefly, microbial DNA was extracted from human and animal stool samples using the QIAamp DNA Stool Mini Kit (QIAGEN, Hilden, Germany). The V3 and V4 regions of the bacteria 16*S* rRNA gene were amplified by polymerase chain reaction (PCR) using primers 338F and 806R (*31*). PCR reactions were performed in triplicate 20-μl mixtures. Primers included an eight-base sequence unique to each sample. Amplicons were extracted from 2% agarose gels and purified using the AxyPrep DNA Gel Extraction Kit (Axygen Biosciences, Union City, CA). Purified amplicons were quantified using QuantiFluor-ST (Promega, USA) and paired-end–sequenced (2 × 250) on an Illumina MiSeq platform according to the standard protocols.

### 16*S* rRNA gene sequence analysis

Raw FASTQ files were demultiplexed and quality-filtered using QIIME (version 1.17; http://qiime.org/). The 250–base pair (bp) reads were truncated at any site of more than three sequential bases receiving an average quality score of <20. Reads shorter than 50 bp, containing ambiguous base calls or barcode/primer errors, were discarded. Chimeric sequences were checked by UCHIME (www.drive5.com/usearch/manual/uchime_algo.html) and removed from subsequent analyses. The remaining high-quality sequences were clustered into OTUs at 97% similarity using USEARCH (version 7.0; www.drive5.com/usearch/manual/). To assess adequacy of sequencing depth, rarefaction analysis was performed using the RDP Rarefaction tool based on the number of sequences and OTUs for each sample. α-Diversity was assessed using the species richness indices (Ace and Chao) and species diversity indices (Shannon) (*12*). Partial least-squares discriminant analysis (PLS-DA) was performed to visually evaluate the difference and similarity of bacterial communities between groups (β-diversity) (*32*). The differentially expressed bacterial taxa between SCZ and HC samples were identified using LEfSe (*33*). Taxonomy was assigned to OTU representatives using the RDP classifier against the SILVA database. A Venn diagram was generated to describe the common and unique OTUs between groups.

### Animal experiments

Animal protocols were approved by the Ethical Committee of Chongqing Medical University (Chongqing, China) and the Third Military Medical University (Chongqing, China). Male GF Kunming mice were bred in the Experimental Animal Research Center at the Third Military Medical University. They were kept in flexible film gnotobiotic isolators until the start of experiments. Mice were fed autoclaved chow and water ad libitum under a 12-hour light/12-hour dark cycle (lights on at 7:30 a.m.) and constant temperature (21° to 22°C) and humidity (55 ± 5%).

### FMT experiments

GF mice were obtained from the Department of Laboratory Animal Science of the Third Military Medical University (Chongqing, China). During the study, the mice were fed autoclaved chow and water ad libitum under a 12-hour light/12-hour dark cycle (lights on at 7:30 a.m.) and constant temperature of 21° to 22°C and humidity of 55 ± 5%. All animal experiments followed the National Institutes of Health *Guide for the Care and Use of Laboratory Animals* and were approved by the Ethics Committee of Chongqing Medical University.

As described previously (*12*, *34*, *35*), fecal samples (i.e., the gut microbiome) from randomly selected subsets of five patients with SCZ (male, aged 26 to 65 years; PANSS scores, 67 to 81) and five HCs (male, aged 31 to 57 years), which were representative of their respective full population distributions (fig. S4A), were used to colonize GF mice (Kunming mice, 5 to 6 weeks old). Briefly, fecal samples were suspended with reduced sterile phosphate-buffered saline (15 ml/g of feces), equal volumes combined for each group, and then these inoculum samples were vortexed for 5 min, followed by 5-min standing to precipitate particles. Each GF mouse was gavaged with 200 μl of fecal suspensions derived from either patients with SCZ or HCs. After colonization, the mice remained in a gnotobiotic environment under the same feeding conditions as before. Last, mice underwent behavioral testing 2 weeks after microbiota transplantation.

### Behavioral tests

#### Open-field test

Mice were placed individually in the corner of an open-field box (*L* × *W* × *H*, 45 cm by 45 cm by 45 cm) and allowed to explore freely for 6 min. Their spontaneous activities over the last 5 min were recorded. The total move distance was designated as an index of locomotor activity, while increased proportion of time or distance spent in the center (inner 25% of the surface area, away from the walls) indexes decreased anxiety.

#### Sociability and social novelty preference test

Mice were placed in a rectangular, three-chambered box. Each chamber was 40 cm by 20 cm by 22 cm (*L* × *W* × *H*) in size. Dividing walls were made from clear plexiglass, with rectangular openings (*L* × *H*, 35 mm by 35 mm) allowing access into each chamber. The test was composed of three sequential 10-min trials: trial 1, habituation (the mouse was allowed to explore the three chambers); trial 2, sociability (an unfamiliar mouse was placed into a mesh wire cage in either the left or right chamber, and exploration of the three chambers by the test mouse was recorded for a further 10 min); and trial 3, social novelty preference (a novel mouse was placed into a mesh wire cage in the chamber opposite the familiar mouse from the previous stage; exploration of the three chambers by the test mouse was again recorded for 10 min). The amount of time spent in each chamber was recorded. An entry was defined as all four paws in one chamber. Test chambers were cleaned with disinfectant and 70% ethanol after each trial.

#### PPI test

The test was measured using the SR-LAB System (San Diego Instruments, San Diego, CA) as described previously. Before testing, each mouse was acclimatized to the plexiglass cylinder with background noise (70 dB) for 5 min. The mouse was then exposed to six blocks of seven trial types that were presented in a pseudorandom order with an average intertrial interval of 15 s. The seven trial types comprised the following: trial 1 (startle-only trial), 40 ms, 120-dB sound burst; trials 2 to 6 (prepulse trials), 120-dB startle stimulus preceded 100 ms earlier by 20-ms prepulse sounds of 74, 78, 82, 86, or 90 dB; and trial 7, 70-dB background noise. The startle response was recorded for 65 ms, measuring every 1 ms from the onset of the startle stimulus. The maximum startle amplitude recorded during the 65-ms sampling window was used as the dependent variable. The percentage of PPI induced by each prepulse intensity was calculated as [1 − (startle amplitude on prepulse trial)/(startle amplitude on pulse alone)] × 100%.

#### Y-maze

The Y-maze apparatus consisted of three dark gray arms (*L* × *W* × *H*, 45 cm by 10 cm by 29 cm). Each mouse was placed at the end of one arm and allowed to freely explore the maze for 8 min. The sequence and total number of arms entered were recorded. Entry into an arm was considered valid only when all four paws of the mouse were inside that arm. The percentage of alternation was used to assess memory performance and calculated by dividing the number of triads containing entries into all three arms by alternation opportunities × 100.

#### Forced swimming test

The mice were placed individually in plexiglass cylinders (30 cm in height and 15 cm in diameter) filled with 15 cm of water (25° ± 1°C). Test sessions lasted for 6 min, with the last 5 min scored for immobility. A mouse was judged to be immobile when it remained floating in an upright position, making only the movements necessary to keep its head above the water.

### Comparisons of metabolite profiles from the FMT model

On week 2 after FMT, the HC microbiota and SCZ microbiota recipient mice were euthanized, and the biological samples including stool, serum, and hippocampus were obtained. Here, three complementary metabolomic methods were chosen to analyze the three types of samples. These detailed metabolomic methods were similar to our previously published literature (*12*). The gas chromatography–mass spectrometry three-dimensional matrices composed of peak indices paired retention time and mass to charge ratio (RT−*m*/*z*), sample names (observations), and normalized peak area percentages were imported into SIMCA-P+14.0 (Umetrics, Umeå, Sweden). The liquid chromatography–mass spectrometry data were collected in both positive and negative electrospray ionization modes. PLS-DA was used to visualize discrimination between the HC microbiota and the SCZ microbiota recipient mice (*36*). By analysis of PLS-DA loadings, the differential metabolites responsible for discriminating between the two groups were identified with variable importance plot values of greater than 1.0 and *P* values of less than 0.05.

Nuclear magnetic resonance data were collected on a 600-MHz spectrometer operating at 599.925-MHz 1H frequency. One-dimensional spin-echo spectra were recorded using Carr-Purcell-Meiboom-Gill relaxation dispersion [recycle delay − 90 − (τ − 180 − τ) *n*]. PLS-DA was used to visualize discrimination between the two groups. The coefficient loading plots of the model were used to identify the spectral variables responsible for sample differentiation on the scores plot. A correlation coefficient (│*r*│) of greater than 0.602 was used as the cutoff value for statistical significance (*36*).

Ingenuity Pathway Analysis (IPA) software (QIAGEN, Redwood City, CA, USA) was used to uncover the predicted molecular pathways and biological functions of differentially expressed metabolites.

### Metagenomic analysis of fecal samples

DNA was extracted from 16 samples using the E.Z.N.A. Stool DNA Kit (Omega Bio-tek, Norcross, GA, USA). The DNA extracted from stool samples was used to construct Illumina paired-end libraries following Illumina’s genomic DNA library preparation procedure. Then, the amplicon library was paired-end–sequenced (2 by 150) on an Illumina HiSeq X platform. Raw FASTQ files were quality-filtered using Trimmomatic (version 0.36) with the following criteria: (i) removal of bases at the start and end of a read below a threshold quality (score < 3) and (ii) the reads were truncated at any site receiving an average quality score of <15 over a 4-bp sliding window, discarding the truncated reads that were shorter than 100 bp.

BMTagger (version 3.102) was used to remove host contamination following the standard operating protocol for removing human sequence, as outlined in the Human Microbiome Project. Read level functional profiling with KO (KEGG Ortholog) was performed using the UProC toolbox (version 2.0.0-rc1) (*37*). The KEGG BRITE databases were used for annotation at the higher functional level. Briefly, the reads from the same KO were binned to REACTION ontology using the KO and REACTION hierarchy relationship, and unique reads were summarized to construct the REACTION ontology count table.

Metaphlan2 (version 2.5.0) and Kaiju (version 1.4.5) with default databases were used to perform the taxonomy composition analysis (*38*). The raw reads count table for several different taxonomy levels from Kaiju classifier was constructed for differential abundance analysis using DESeq2. R package DESeq2 (version 1.10.1) was used to detect the differential abundance ontologies or taxonomy catalog (*39*). The de bruijn graph–based assembler MEGAHIT (version 1.0.6) was used to assemble short reads. The contigs with length greater than 500 bp were used to make the reference index for bowtie alignment, the unaligned reads were collected to co-assemble with the same assembly parameter, and the minimal contig length was set to 200. MetaProdigal (version 2.6.3) was used for gene prediction of metagenomic contigs (*40*). The abundance of each nonredundant gene [measured as transcripts-per-million (TPM)] was quantified with kallisto (version 0.42.5). The function space of the nonredundant gene was annotated with KEGG.

### Statistical analysis

Statistical analyses were carried out using SPSS version 18 (SPSS, Chicago, IL, USA). All continuous variables such as bacterial α-diversity, behavioral data, age, and body mass index were presented as means ± SEM, unless otherwise indicated and compared between groups using Student’s *t* test. Categorical data (sex) were analyzed by χ^2^ test. Statistical significance level was set at *P* < 0.05.

## Supplementary Material

http://advances.sciencemag.org/cgi/content/full/5/2/eaau8317/DC1

## SUPPLEMENTARY MATERIALS

Supplementary material for this article is available at http://advances.sciencemag.org/cgi/content/full/5/2/eaau8317/DC1 Fig. S1. Gut microbial composition differences between patients with SCZ and HC subjects. Fig. S2. Impact of confounding variables on global gut microbial phenotypes. Fig. S3. Comparison of microbial markers between patients with SCZ and major depressive disorder. Fig. S4. Comparison of gut microbial characteristics between SCZ microbiota and HC microbiota recipient mice. Fig. S5. Metabolomic analysis of fecal, serum, and hippocampal samples obtained from SCZ microbiota and HC microbiota recipient mice. Fig. S6. IPA shows that differentially expressed fecal, serum, and hippocampus metabolites were consistently involved in amino acid metabolism, especially glutamate metabolism. Fig. S7. Levels of glutamine, glutamic acid, and GABA in SCZ microbiota and HC microbiota recipient mice. Table S1. Detailed clinical characteristics of participants. Table S2A. Discriminatory OTUs between patients with SCZ and HC subjects. Table S2B. Discriminatory KEGG pathways between SCZ microbiota and HC microbiota recipient mice. Table S3A. Differential fecal metabolites between SCZ microbiota and HC microbiota recipient mice. Table S3B. Differential serum metabolites between SCZ microbiota and HC microbiota recipient mice. Table S3C. Differential hippocampus metabolites between SCZ microbiota and HC microbiota recipient mice.

## References

[1] Long J., Huang G., Liang W., Liang B., Chen Q., Xie J., Jiang J., Su L., The prevalence of schizophrenia in mainland China: Evidence from epidemiological surveys. Acta Psychiatr. Scand. 130, 244–256 (2014). 24916190 10.1111/acps.12296

[2] The Schizophrenia Psychiatric Genome-Wide Association Study (GWAS) Consortium, Genome-wide association study identifies five new schizophrenia loci. Nat. Genet. 43, 969–976 (2011). 21926974 10.1038/ng.940 PMC3303194

[3] Schizophrenia Working Group of the Psychiatric Genomics Consortium, Biological insights from 108 schizophrenia-associated genetic loci. Nature 511, 421–427 (2014). 25056061 10.1038/nature13595 PMC4112379

[4] The Human Microbiome Project Consortium, Structure, function and diversity of the healthy human microbiome. Nature 486, 207–214 (2012). 22699609 10.1038/nature11234 PMC3564958

[5] Cryan J. F., Dinan T. G., Mind-altering microorganisms: The impact of the gut microbiota on brain and behaviour. Nat. Rev. Neurosci. 13, 701–712 (2012). 22968153 10.1038/nrn3346

[6] Hsiao E. Y., McBride S. W., Hsien S., Sharon G., Hyde E. R., McCue T., Codelli J. A., Chow J., Reisman S. E., Petrosino J. F., Patterson P. H., Mazmanian S. K., Microbiota modulate behavioral and physiological abnormalities associated with neurodevelopmental disorders. Cell 155, 1451–1463 (2013). 24315484 10.1016/j.cell.2013.11.024 PMC3897394

[7] Diaz Heijtz R., Wang S., Anuar F., Qian Y., Björkholm B., Samuelsson A., Hibberd M. L., Forssberg H., Pettersson S., Normal gut microbiota modulates brain development and behavior. Proc. Natl. Acad. Sci. U.S.A. 108, 3047–3052 (2011). 21282636 10.1073/pnas.1010529108 PMC3041077

[8] Gareau M. G., Wine E., Rodrigues D. M., Cho J. H., Whary M. T., Philpott D. J., MacQueen G., Sherman P. M., Bacterial infection causes stress-induced memory dysfunction in mice. Gut 60, 307–317 (2011). 20966022 10.1136/gut.2009.202515

[9] Desbonnet L., Clarke G., Shanahan F., Dinan T. G., Cryan J. F., Microbiota is essential for social development in the mouse. Mol. Psychiatry 19, 146–148 (2014). 23689536 10.1038/mp.2013.65 PMC3903109

[10] Sampson T. R., Debelius J. W., Thron T., Janssen S., Shastri G. G., Ilhan Z. E., Challis C., Schretter C. E., Rocha S., Gradinaru V., Chesselet M.-F., Keshavarzian A., Shannon K. M., Krajmalnik-Brown R., Wittung-Stafshede P., Knight R., Mazmanian S. K., Gut microbiota regulate motor deficits and neuroinflammation in a model of Parkinson’s disease. Cell 167, 1469–1480.e12 (2016). 27912057 10.1016/j.cell.2016.11.018 PMC5718049

[11] Zeng L., Zeng B., Wang H., Li B., Huo R., Zheng P., Zhang X., Du X., Liu M., Fang Z., Xu X., Zhou C., Chen J., Li W., Guo J., Wei H., Xie P., Microbiota modulates behavior and protein kinase C mediated cAMP response element-binding protein signaling. Sci. Rep. 6, 29998 (2016). 27444685 10.1038/srep29998 PMC4956747

[12] Zheng P., Zeng B., Zhou C., Liu M., Fang Z., Xu X., Zeng L., Chen J., Fan S., Du X., Zhang X., Yang D., Yang Y., Meng H., Li W., Melgiri N. D., Licinio J., Wei H., Xie P., Gut microbiome remodeling induces depressive-like behaviors through a pathway mediated by the host’s metabolism. Mol. Psychiatry 21, 786–796 (2016). 27067014 10.1038/mp.2016.44

[13] Lv F., Chen S., Wang L., Jiang R., Tian H., Li J., Yao Y., Zhuo C., The role of microbiota in the pathogenesis of schizophrenia and major depressive disorder and the possibility of targeting microbiota as a treatment option. Oncotarget 8, 100899–100907 (2017). 29246029 10.18632/oncotarget.21284 PMC5725071

[14] Babulas V., Factor-Litvak P., Goetz R., Schaefer C. A., Brown A. S., Prenatal exposure to maternal genital and reproductive infections and adult schizophrenia. Am. J. Psychiatry 163, 927–929 (2006). 16648337 10.1176/ajp.2006.163.5.927

[15] Fadgyas-Stanculete M., Buga A.-M., Popa-Wagner A., Dumitrascu D. L., The relationship between irritable bowel syndrome and psychiatric disorders: From molecular changes to clinical manifestations. J. Mol. Psychiatry 2, 4 (2014). 25408914 10.1186/2049-9256-2-4 PMC4223878

[16] Clarke G., Grenham S., Scully P., Fitzgerald P., Moloney R. D., Shanahan F., Dinan T. G., Cryan J. F., The microbiome-gut-brain axis during early life regulates the hippocampal serotonergic system in a sex-dependent manner. Mol. Psychiatry 18, 666–673 (2013). 22688187 10.1038/mp.2012.77

[17] Miller B. J., Buckley P., Seabolt W., Mellor A., Kirkpatrick B., Meta-analysis of cytokine alterations in schizophrenia: Clinical status and antipsychotic effects. Biol. Psychiatry 70, 663–671 (2011). 21641581 10.1016/j.biopsych.2011.04.013 PMC4071300

[18] Goff D. C., Coyle J. T., The emerging role of glutamate in the pathophysiology and treatment of schizophrenia. Am. J. Psychiatry 158, 1367–1377 (2001). 11532718 10.1176/appi.ajp.158.9.1367

[19] Lutkenhoff E. S., van Erp T. G., Thomas M. A., Therman S., Manninen M., Huttunen M. O., Kaprio J., Lönnqvist J., O’Neill J., Cannon T. D., Proton MRS in twin pairs discordant for schizophrenia. Mol. Psychiatry 15, 308–318 (2010). 18645571 10.1038/mp.2008.87

[20] Stan A. D., Ghose S., Zhao C., Hulsey K., Mihalakos P., Yanagi M., Morris S. U., Bartko J. J., Choi C., Tamminga C. A., Magnetic resonance spectroscopy and tissue protein concentrations together suggest lower glutamate signaling in dentate gyrus in schizophrenia. Mol. Psychiatry 20, 433–439 (2015). 24912493 10.1038/mp.2014.54

[21] Jones C. A., Watson D. J. G., Fone K. C. F., Animal models of schizophrenia. Br. J. Pharmacol. 164, 1162–1194 (2011). 21449915 10.1111/j.1476-5381.2011.01386.x PMC3229756

[22] Karlsgodt K. H., Robleto K., Trantham-Davidson H., Jairl C., Cannon T. D., Lavin A., Jentsch J. D., Reduced dysbindin expression mediates N-methyl-D-aspartate receptor hypofunction and impaired working memory performance. Biol. Psychiatry 69, 28–34 (2011). 21035792 10.1016/j.biopsych.2010.09.012 PMC4204919

[23] Nilsson M., Waters S., Waters N., Carlsson A., Carlsson M. L., A behavioural pattern analysis of hypoglutamatergic mice—Effects of four different antipsychotic agents. J. Neural Transm. 108, 1181–1196 (2001). 11725821 10.1007/s007020170008

[24] Procaccini C., Aitta-aho T., Jaako-Movits K., Zharkovsky A., Panhelainen A., Sprengel R., Linden A.-M., Korpi E. R., Excessive novelty-induced c-Fos expression and altered neurogenesis in the hippocampus of GluA1 knockout mice. Eur. J. Neurosci. 33, 161–174 (2011). 21073553 10.1111/j.1460-9568.2010.07485.x

[25] Sharon G., Sampson T. R., Geschwind D. H., Mazmanian S. K., The central nervous system and the gut microbiome. Cell 167, 915–932 (2016). 27814521 10.1016/j.cell.2016.10.027 PMC5127403

[26] Dinan T. G., Cryan J. F., Gut instincts: Microbiota as a key regulator of brain development, ageing and neurodegeneration. J. Physiol. 595, 489–503 (2017). 27641441 10.1113/JP273106 PMC5233671

[27] Zhang L., Zhao J., Profile of minocycline and its potential in the treatment of schizophrenia. Neuropsychiatr. Dis. Treat. 10, 1103–1111 (2014). 24971013 10.2147/NDT.S64236 PMC4069141

[28] Tessier C., Sweers K., Frajerman A., Bergaoui H., Ferreri F., Delva C., Lapidus N., Lamaziere A., Roiser J. P., De Hert M., Nuss P., Membrane lipidomics in schizophrenia patients: A correlational study with clinical and cognitive manifestations. Transl. Psychiatry 6, e906 (2016). 27701405 10.1038/tp.2016.142 PMC5315538

[29] Romme I. A. C., de Reus M. A., Ophoff R. A., Kahn R. S., van den Heuvel M. P., Connectome disconnectivity and cortical gene expression in patients with schizophrenia. Biol. Psychiatry 81, 495–502 (2017). 27720199 10.1016/j.biopsych.2016.07.012

[30] Yang J., Chen T., Sun L., Zhao Z., Qi X., Zhou K., Cao Y., Wang X., Qiu Y., Su M., Zhao A., Wang P., Yang P., Wu J., Feng G., He L., Jia W., Wan C., Potential metabolite markers of schizophrenia. Mol. Psychiatry 18, 67–78 (2013). 22024767 10.1038/mp.2011.131 PMC3526727

[31] Bruce-Keller A. J., Salbaum J. M., Luo M., Blanchard E. IV, Taylor C. M., Welsh D. A., Berthoud H.-R., Obese-type gut microbiota induce neurobehavioral changes in the absence of obesity. Biol. Psychiatry 77, 607–615 (2015). 25173628 10.1016/j.biopsych.2014.07.012 PMC4297748

[32] Newell C., Bomhof M. R., Reimer R. A., Hittel D. S., Rho J. M., Shearer J., Ketogenic diet modifies the gut microbiota in a murine model of autism spectrum disorder. Mol. Autism 7, 37 (2016). 27594980 10.1186/s13229-016-0099-3 PMC5009541

[33] Segata N., Izard J., Waldron L., Gevers D., Miropolsky L., Garrett W. S., Huttenhower C., Metagenomic biomarker discovery and explanation. Genome Biol. 12, R60 (2011). 21702898 10.1186/gb-2011-12-6-r60 PMC3218848

[34] Wong S. H., Zhao L., Zhang X., Nakatsu G., Han J., Xu W., Xiao X., Kwong T. N. Y., Tsoi H., Wu W. K. K., Zeng B., Chan F. K. L., Sung J. J. Y., Wei H., Yu J., Gavage of fecal samples from patients with colorectal cancer promotes intestinal carcinogenesis in germ-free and conventional mice. Gastroenterology 153, 1621–1633.e6 (2017). 28823860 10.1053/j.gastro.2017.08.022

[35] Koren O., Goodrich J. K., Cullender T. C., Spor A., Laitinen K., Bäckhed H. K., Gonzalez A., Werner J. J., Angenent L. T., Knight R., Bäckhed F., Isolauri E., Salminen S., Ley R. E., Host remodeling of the gut microbiome and metabolic changes during pregnancy. Cell 150, 470–480 (2012). 22863002 10.1016/j.cell.2012.07.008 PMC3505857

[36] Zheng P., Gao H. C., Li Q., Shao W. H., Zhang M. L., Cheng K., Yang D. Y., Fan S. H., Chen L., Fang L., Xie P., Plasma metabonomics as a novel diagnostic approach for major depressive disorder. J. Proteome Res. 11, 1741–1748 (2012). 22239730 10.1021/pr2010082

[37] Kanehisa M., Furumichi M., Tanabe M., Sato Y., Morishima K., KEGG: New perspectives on genomes, pathways, diseases and drugs. Nucleic Acids Res. 45, D353–D361 (2017). 27899662 10.1093/nar/gkw1092 PMC5210567

[38] Menzel P., Ng K. L., Krogh A., Fast and sensitive taxonomic classification for metagenomics with Kaiju. Nat. Commun. 7, 11257 (2016). 27071849 10.1038/ncomms11257 PMC4833860

[39] Love M. I., Huber W., Anders S., Moderated estimation of fold change and dispersion for RNA-seq data with DESeq2. Genome Biol. 15, 550 (2014). 25516281 10.1186/s13059-014-0550-8 PMC4302049

[40] Hyatt D., LoCascio P. F., Hauser L. J., Uberbacher E. C., Gene and translation initiation site prediction in metagenomic sequences. Bioinformatics 28, 2223–2230 (2012). 22796954 10.1093/bioinformatics/bts429

