# Supplementary material for: The gut microbiome from patients with schizophrenia modulates the glutamate-glutamine-GABA cycle and schizophrenia-relevant behaviors in mice
Source: Sci Adv. 2019 Feb 6;5(2):eaau8317. doi: 10.1126/sciadv.aau8317 (PMC6365110; doi:10.1126/sciadv.aau8317)
Supplement: http://advances.sciencemag.org/cgi/content/full/5/2/eaau8317/DC1 [file supp_5_2_eaau8317__index.html]

Science Advances | Science Advances

## Supplementary Materials

**This PDF file includes:**

- Fig. S1. Gut microbial composition differences between patients with SCZ and HC subjects.
- Fig. S2. Impact of confounding variables on global gut microbial phenotypes.
- Fig. S3. Comparison of microbial markers between patients with SCZ and major depressive disorder.
- Fig. S4. Comparison of gut microbial characteristics between SCZ microbiota and HC microbiota recipient mice.
- Fig. S5. Metabolomic analysis of fecal, serum, and hippocampal samples obtained from SCZ microbiota and HC microbiota recipient mice.
- Fig. S6. IPA shows that differentially expressed fecal, serum, and hippocampus metabolites were consistently involved in amino acid metabolism, especially glutamate metabolism.
- Fig. S7. Levels of glutamine, glutamic acid, and GABA in SCZ microbiota and HC microbiota recipient mice.
- Table S1. Detailed clinical characteristics of participants.
- Table S2A. Discriminatory OTUs between patients with SCZ and HC subjects.
- Table S2B. Discriminatory KEGG pathways between SCZ microbiota and HC microbiota recipient mice.
- Table S3A. Differential fecal metabolites between SCZ microbiota and HC microbiota recipient mice.
- Table S3B. Differential serum metabolites between SCZ microbiota and HC microbiota recipient mice.
- Table S3C. Differential hippocampus metabolites between SCZ microbiota and HC microbiota recipient mice.

Download PDF

**Files in this Data Supplement:**

- Adobe PDF - aau8317\_SM.pdf
