# Supplementary material for: The gut microbiome from patients with schizophrenia modulates the glutamate-glutamine-GABA cycle and schizophrenia-relevant behaviors in mice
Source: Sci Adv. 2019 Feb 6;5(2):eaau8317. doi: 10.1126/sciadv.aau8317 (PMC6365110; doi:10.1126/sciadv.aau8317)
Supplement: http://advances.sciencemag.org/cgi/content/full/5/2/eaau8317/DC1 [file aau8317_SM.pdf]

Science Advances
AAAS

advances.sciencemag.org/cgi/content/full/5/2/eaau8317/DC1

Supplementary Materials for

The gut microbiome from patients with schizophrenia modulates the glutamate-glutamine-GABA cycle and schizophrenia-relevant behaviors in mice

Peng Zheng, Benhua Zeng, Meiling Liu, Jianjun Chen, Junxi Pan, Yu Han, Yiyun Liu, Ke Cheng, Chanjuan Zhou, Haiyang Wang, Xinyu Zhou, Siwen Gui, Seth W. Perry, Ma-Li Wong, Julio Licinio*, Hong Wei*, Peng Xie*

*Corresponding author. Email: xiepeng@cqmu.edu.cn (P.X.); weihong63528@163.com (H.W.); licinioj@upstate.edu (J.L.)

Published 6 February 2019, *Sci. Adv.* **5**, eaau8317 (2019)
DOI: 10.1126/sciadv.aau8317

This PDF file includes:

Fig. S1. Gut microbial composition differences between patients with SCZ and HC subjects.
Fig. S2. Impact of confounding variables on global gut microbial phenotypes.
Fig. S3. Comparison of microbial markers between patients with SCZ and major depressive disorder.
Fig. S4. Comparison of gut microbial characteristics between SCZ microbiota and HC microbiota recipient mice.
Fig. S5. Metabolomic analysis of fecal, serum, and hippocampal samples obtained from SCZ microbiota and HC microbiota recipient mice.
Fig. S6. IPA shows that differentially expressed fecal, serum, and hippocampus metabolites were consistently involved in amino acid metabolism, especially glutamate metabolism.
Fig. S7. Levels of glutamine, glutamic acid, and GABA in SCZ microbiota and HC microbiota recipient mice.
Table S1. Detailed clinical characteristics of participants.
Table S2A. Discriminatory OTUs between patients with SCZ and HC subjects.
Table S2B. Discriminatory KEGG pathways between SCZ microbiota and HC microbiota recipient mice.
Table S3A. Differential fecal metabolites between SCZ microbiota and HC microbiota recipient mice.
Table S3B. Differential serum metabolites between SCZ microbiota and HC microbiota recipient mice.
Table S3C. Differential hippocampus metabolites between SCZ microbiota and HC microbiota recipient mice.

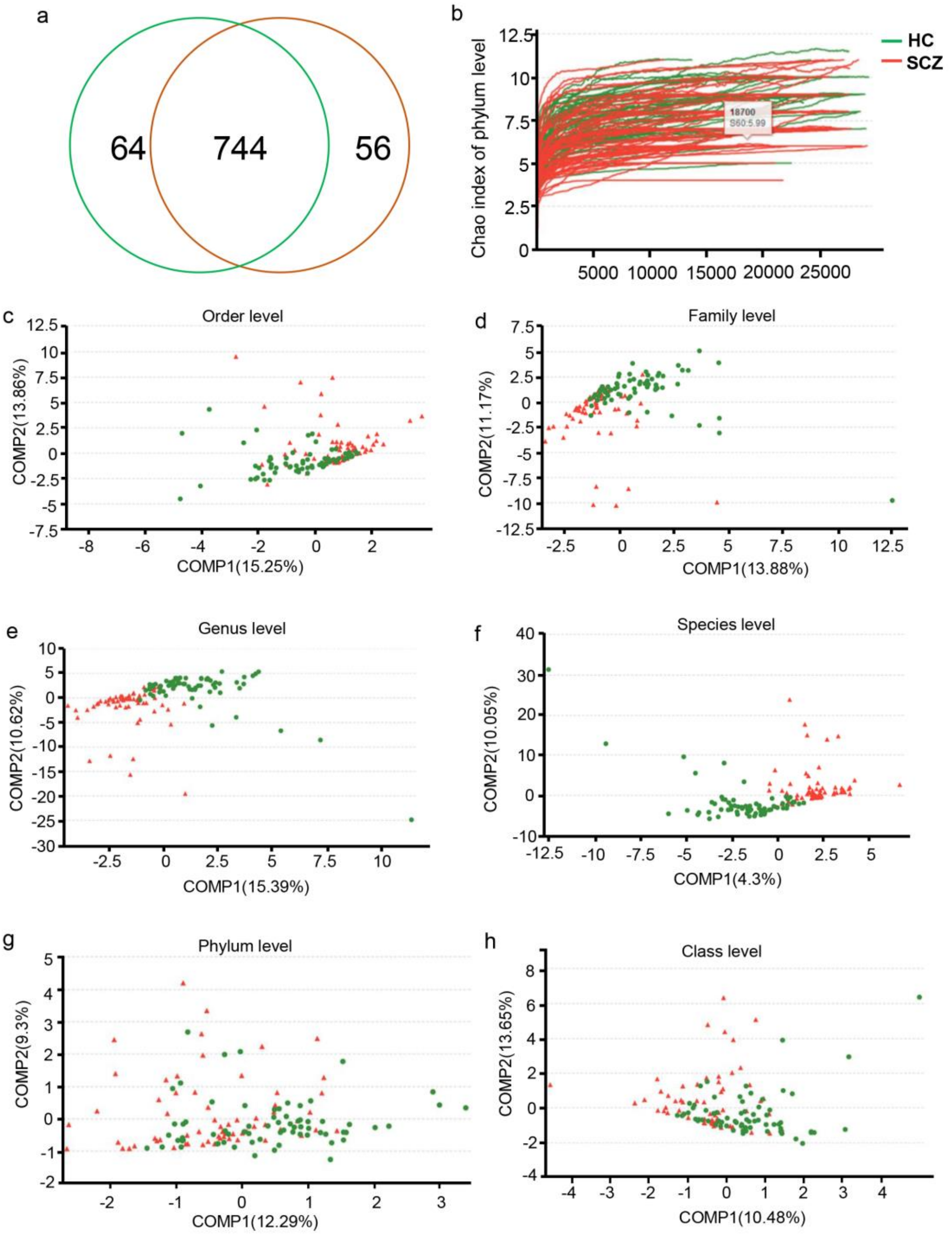

Fig. S1. Gut microbial composition differences between patients with SCZ and HC subjects. (**a**) A Venn diagram showed that 744 of 864 OTUs were detected in the two groups, while 56 and 64 OTUs were unique to SCZ and HC subjects, respectively. (**b**) The majority of rarefaction curves tended to approach the saturation plateau, suggesting the sequencing depth of the gut microbiome was sufficient. (**c-f**) The microbial composition of SCZ was significantly different from that in HCs from order to OTU levels (n=69, HC; n=63, SCZ). (**g-h**) A discriminative trend of gut microbial composition between SCZ and HCs was observed at the phylum or class levels (n=69, HC; n=63, SCZ).

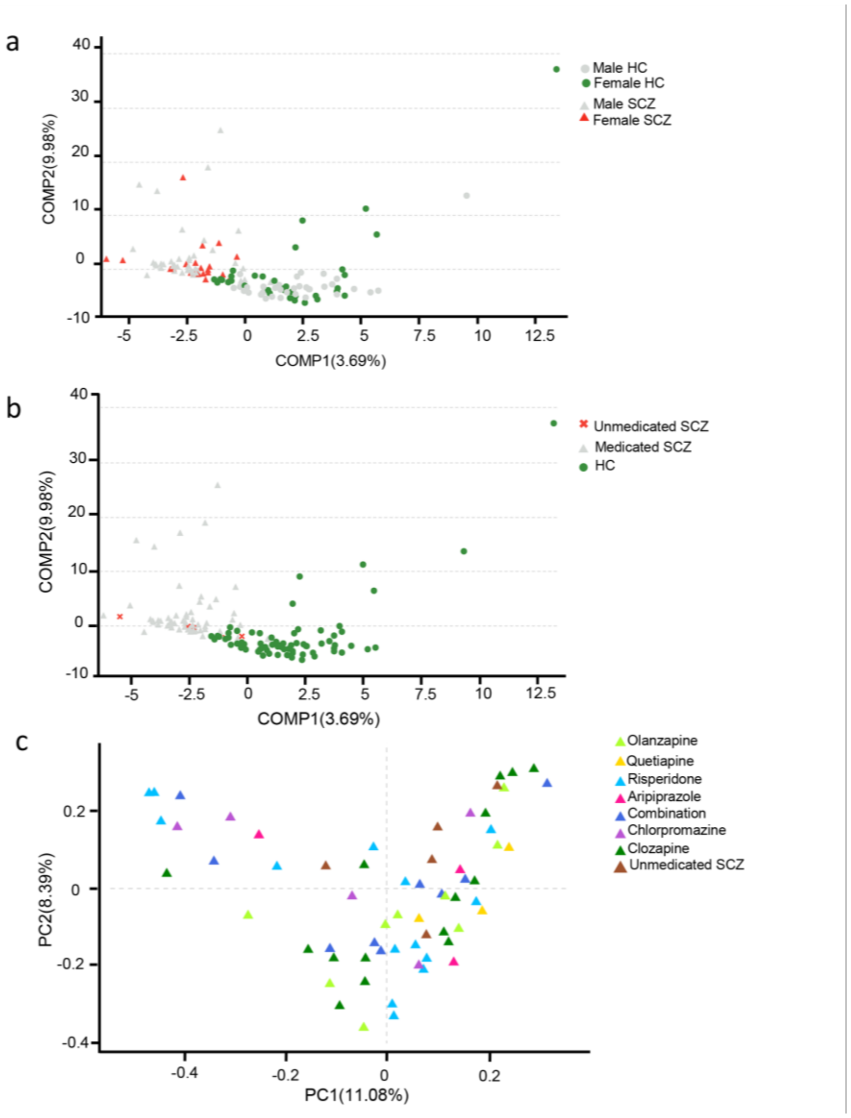

Fig. S2. Impact of confounding variables on global gut microbial phenotypes. The global microbial phenotypes were not clustered based on (**a**) sex, (**b**) antipsychotic use, or (**c**) antipsychotic type.

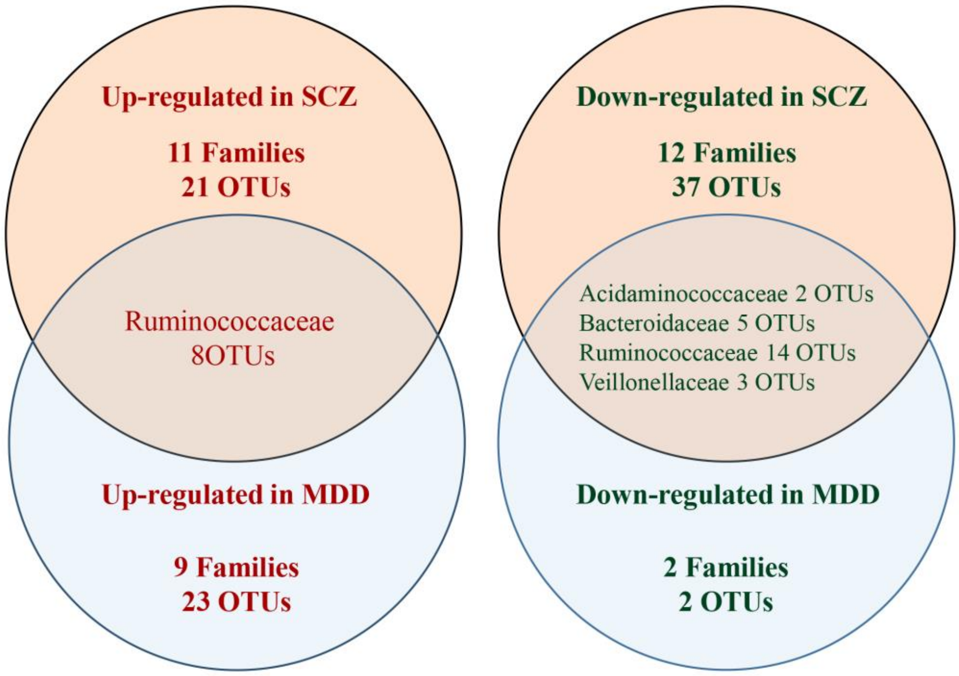

Fig. S3. Comparison of microbial markers between patients with SCZ and major depressive disorder. Venn diagram showing that only four microbes including Ruminococcaceae, Acidaminococcaceae, Bacteroidaceae, and Veillonellaceae were synchronous in both SCZ and major depressive disorder (MDD).

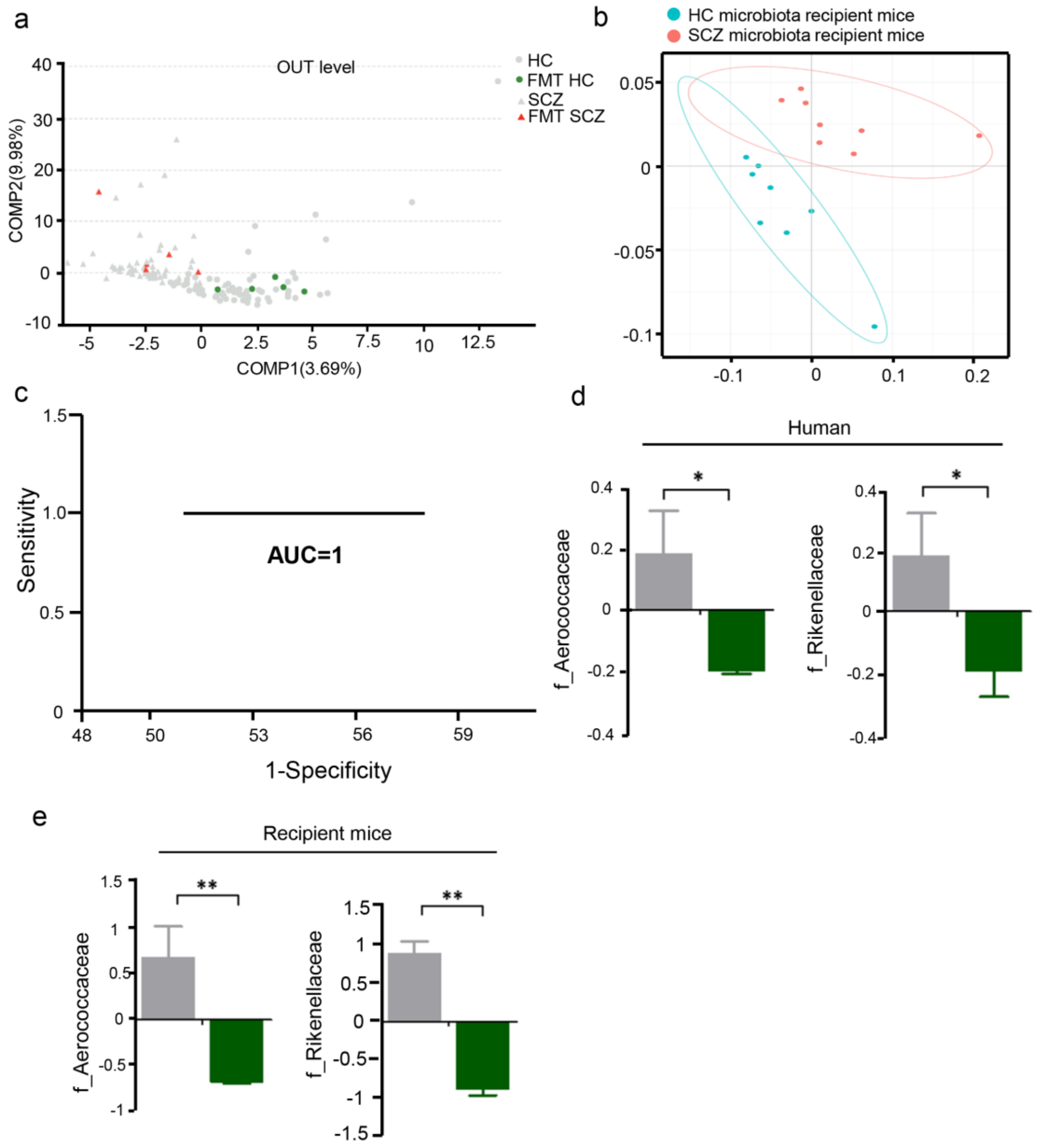

Fig. S4. Comparison of gut microbial characteristics between SCZ microbiota and HC microbiota recipient mice. (**a**) Gut microbial phenotypes of SCZ subjects and HC donors were similar to their corresponding groups. The samples used for fecal microbiota transplantation experiments (n=5 for each group) were representative of their respective populations (n=64, HC; n=58, SCZ); (**b**) Significant differences were found between the gut microbiotic community compositions of 'SCZ microbiota' recipient mice and 'HC microbiota' recipient mice (n=8 for each group); (**c**) The combination of Aerococcaceae and Rikenellaceae completely discriminated 'SCZ microbiota' recipient mice from their HC counterparts with an AUC of 1 (i.e. with 100% accuracy) (n=8 for each group); (**d-e**) The key discriminative microbial markers of SCZ subjects were successfully colonized in the 'SCZ microbiota' recipient mice. Comparison of relative abundance of microbial markers. Identical changes in Aerococcaceae and Rikenellaceae composition were seen in both (d) human SCZ subjects (n=69, HC; n=63, SCZ) and in (e) 'SCZ microbiota' recipient mice (n=8 for each group). All data were presented as mean ± SEM. *p<0.05, **p<0.01.

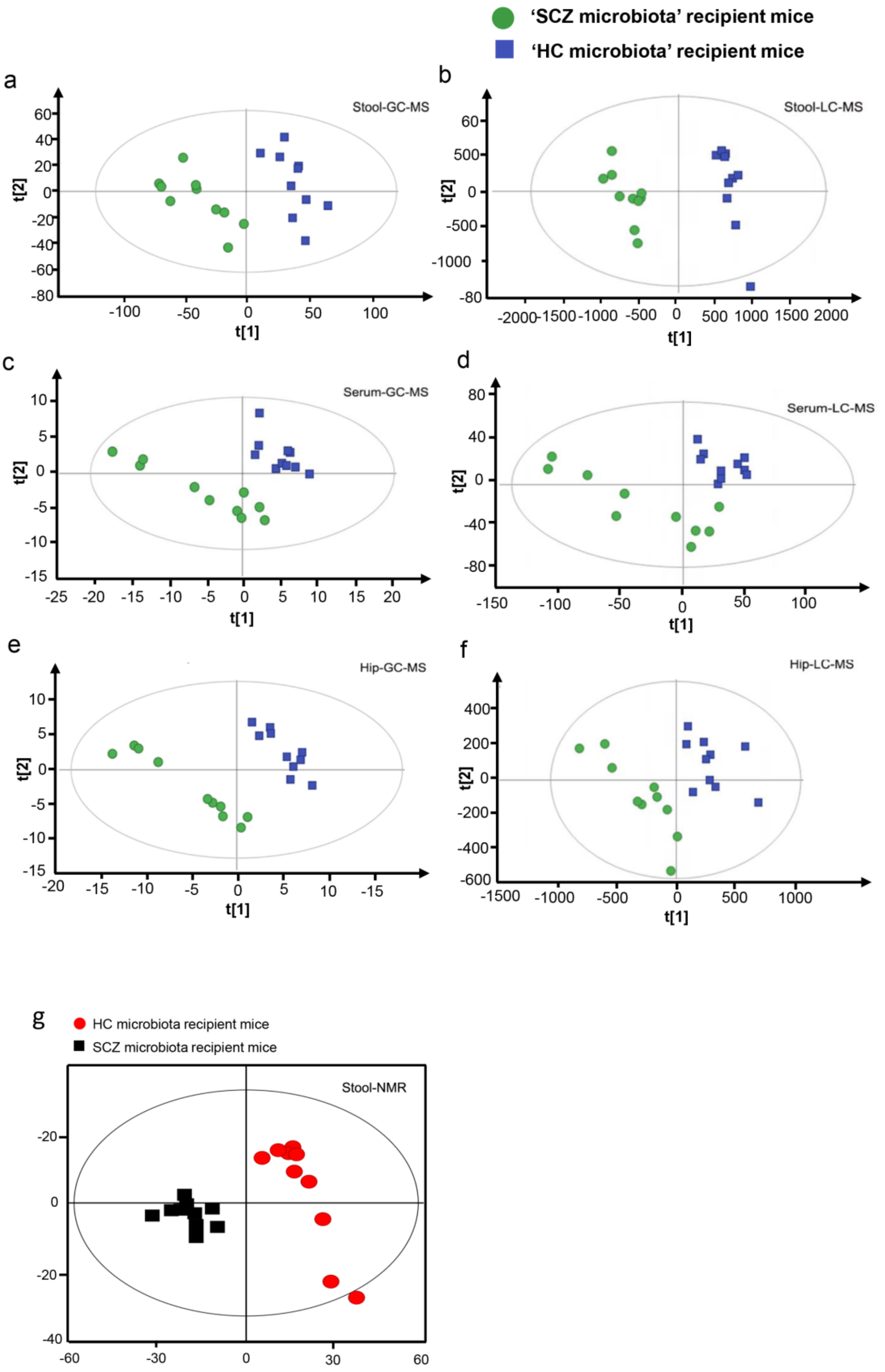

Fig. S5. Metabolomic analysis of fecal, serum, and hippocampal samples obtained from SCZ microbiota and HC microbiota recipient mice. (**a-f**) Gas chromatography and liquid mass spectrometry (GC-MS and LC-MS) as well as (**g**) Nuclear Magnetic Resonance (NMR) based

metabolomic analyses demonstrate that the metabolic signatures of fecal, serum, and hippocampal samples obtained from ‘SCZ microbiota’ recipient mice and ‘HC microbiota’ recipient mice are significantly different.

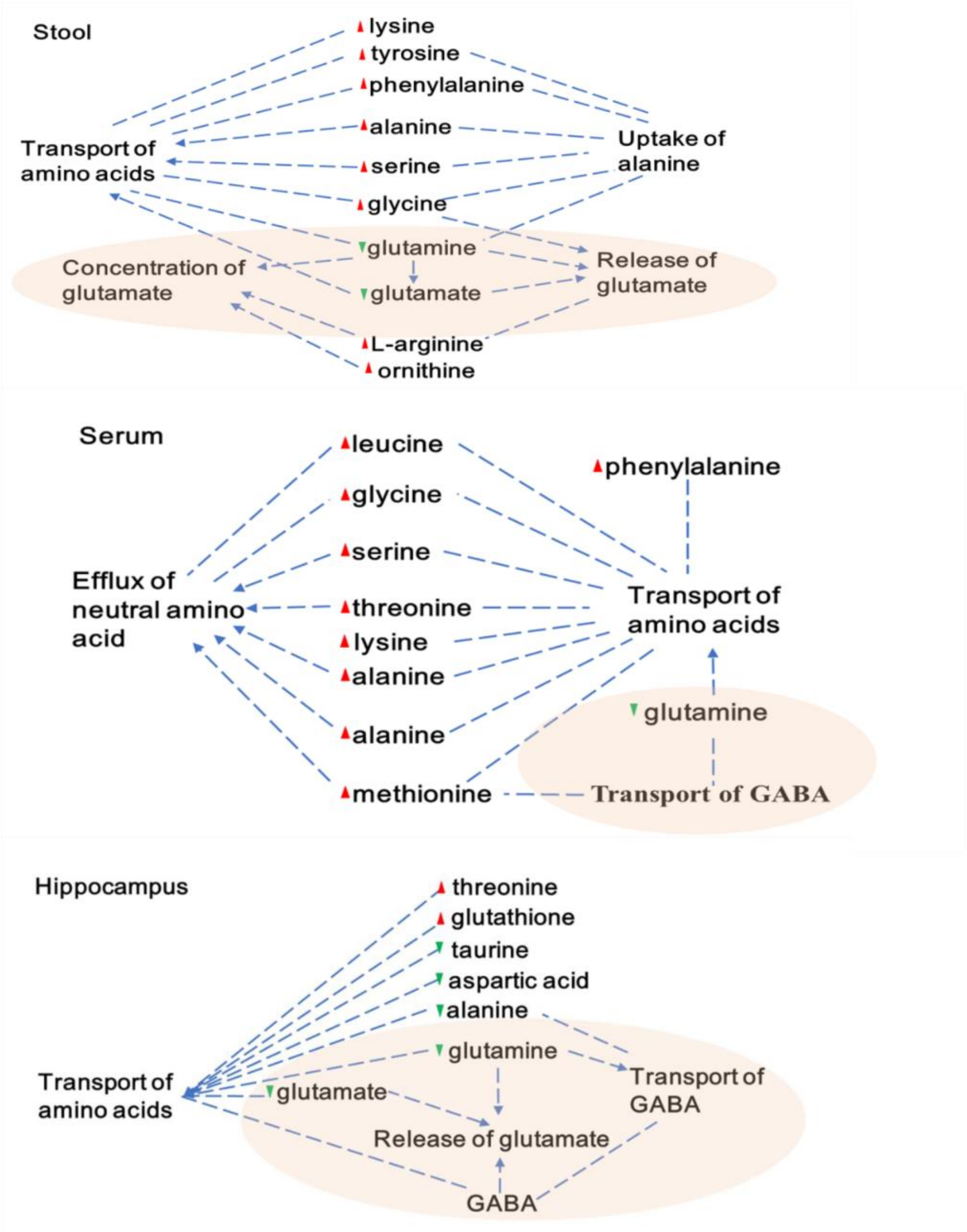

Fig. S6. IPA shows that differentially expressed fecal, serum, and hippocampus metabolites were consistently involved in amino acid metabolism, especially glutamate metabolism. IPA analysis showed that 'SCZ microbiota' recipient mice were characterized by alterations in the gut-brain glutamate-glutamine-GABA cycle. Red triangles indicate metabolites that were increased in 'SCZ microbiota' recipient mice relative to 'HC microbiota' recipient mice (n=10 each group). Green triangles indicate metabolites that were decreased in 'SCZ microbiota' recipient mice.

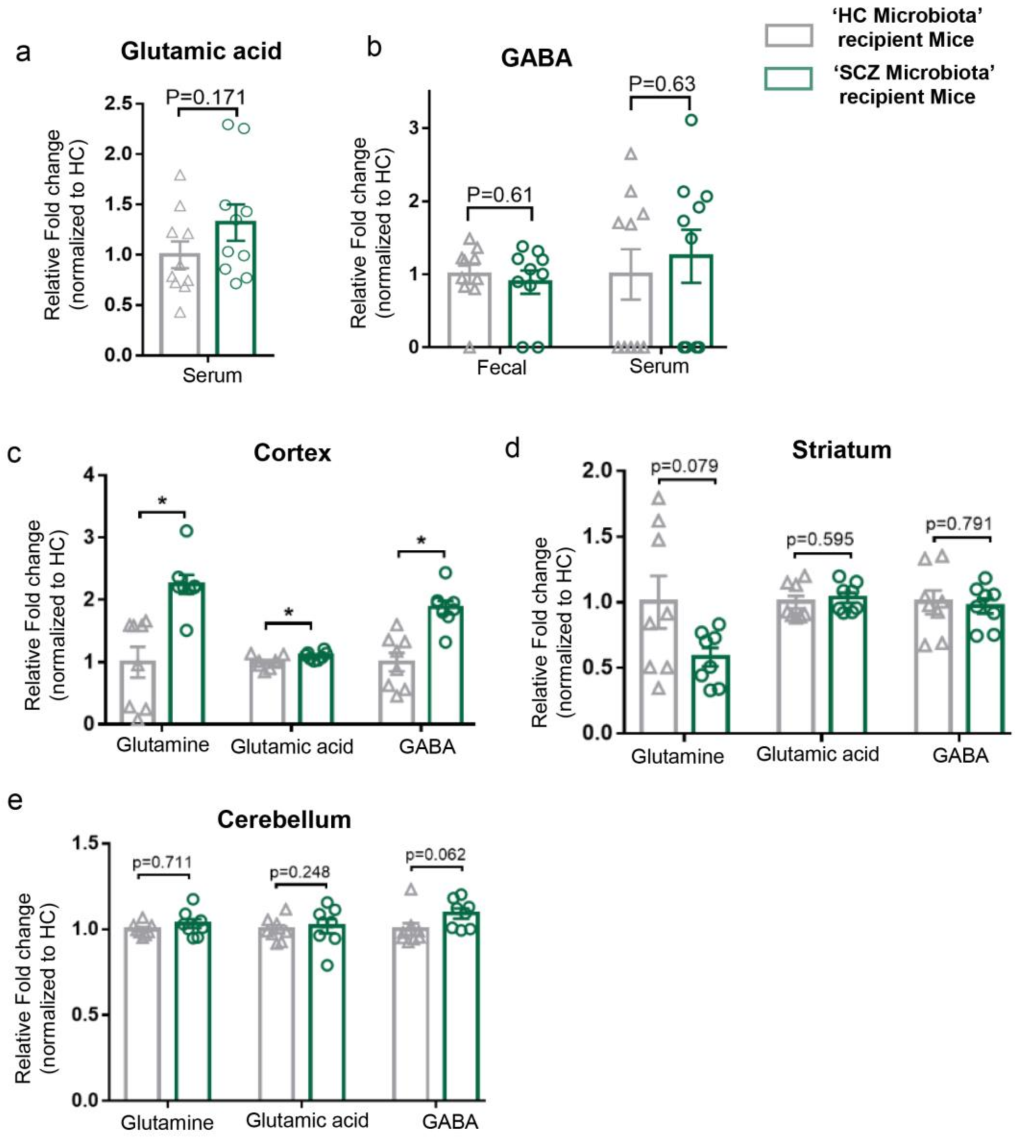

Fig. S7. Levels of glutamine, glutamic acid, and GABA in SCZ microbiota and HC microbiota recipient mice. (**a-b**) Glutamate in serum, and GABA in feces and serum, were not significantly changed in 'SCZ Microbiota' versus 'HC Microbiota' recipient mice. (**c**) In cortex, glutamine, glutamate, and GABA were increased in 'SCZ Microbiota' mice relative to 'HC Microbiota' recipient mice. (**d-e**) In the striatum and cerebellum, glutamine, glutamate, and GABA were not significantly different between 'SCZ Microbiota' and 'HC Microbiota'.

Table S1. Detailed clinical characteristics of participants.

	Cohort 1			*Cohort 2*		
Variables [a]	SCZ	HC	*P*	MDD	HC	P [b]
Sample Size	63	69	-	58	63	-
Sex (M/F)	42/21	36/33	0.091	22/36	23/40	0.87
Age (year)	43.49±1.68	39.99±1.62	0.135	40.6±11.7	41.8±12.3	0.59
BMI	22.90±0.32	23.16±0.33	0.585	22.0±2.4	22.6±2.5	0.19
Medication (Y/N)	58/5	-	-	19/39	-	-
PANSS	71.87±1.85	-	-	-	-	-
Separate/combination therapy	49/9	-	-	-	-	-
HDRS Scores	-	-	-	22.8±4.4	0.3±0.7	0.00

[a] Two-tailed student test for continuous variables (age, BMI, and HDRS); Chi-square analyses for categorical variables (sex); values are expressed as the mean ± standard error of the mean; SCZ, schizophrenia subjects; MDD, major depressive disorder; HC, healthy controls; M/F, male/female; Y/N, yes/no; BMI, body mass index; PANSS, Positive and Negative Syndrome Scale Score; HDRS, Hamilton Depression Rating Scale.

Table S2A. Discriminatory OTUs between patients with SCZ and HC subjects.

OTUs	SCZ Relative to HCs	Taxonomic Assignment	Discriminative OTUs at the family level
OTU258	Down	p__Firmicutes; c__Negativicutes; o__Selenomonadales; f__Acidaminococcaceae; g__Phascolarctobacterium; s__unclassified_g__Phascolarctobacterium	f__Acidaminococcaceae
OTU77	Down	p__Bacteroidetes; c__Bacteroidia; o__Bacteroidales; f__Bacteroidaceae; g__Bacteroides; s__Bacteroides_eggerthii	f__Bacteroidaceae
OTU400	Down	p__Bacteroidetes; c__Bacteroidia; o__Bacteroidales; f__Bacteroidaceae; g__Bacteroides; s__Bacteroides_massiliensis	f__Bacteroidaceae
OTU92	Down	p__Bacteroidetes; c__Bacteroidia; o__Bacteroidales; f__Bacteroidaceae; g__Bacteroides; s__unclassified_g__Bacteroides	f__Bacteroidaceae
OTU700	Down	p__Firmicutes; c__Bacilli; o__Lactobacillales; f__Carnobacteriaceae; g__Granulicatella; s__uncultured_organism_g__Granulicatella	f__Carnobacteriaceae
OTU498	Down	p__Actinobacteria; c__Actinobacteria; o__Coriobacteriales; f__Coriobacteriaceae; g__Collinsella; s__Collinsella_stercoris	f__Coriobacteriaceae
OTU26	Down	p__Proteobacteria; c__Gammaproteobacteria; o__Enterobacteriales; f__Enterobacteriaceae; g__Citrobacter; s__unclassified_g__Citrobacter	f__Enterobacteriaceae
OTU418	Down	p__Proteobacteria; c__Gammaproteobacteria; o__Enterobacteriales; f__Enterobacteriaceae; g__Hafnia; s__uncultured_organism_g__Hafnia	f__Enterobacteriaceae
OTU224	Down	p__Proteobacteria; c__Gammaproteobacteria; o__Enterobacteriales; f__Enterobacteriaceae; g__Klebsiella; s__unclassified_g__Klebsiella	f__Enterobacteriaceae
OTU503	Down	p__Proteobacteria; c__Gammaproteobacteria; o__Enterobacteriales; f__Enterobacteriaceae; g__Raoultella; s__Raoultella_ornithinolytica_B6	f__Enterobacteriaceae
OTU149	Down	p__Firmicutes; c__Erysipelotrichia; o__Erysipelotrichales; f__Erysipelotrichaceae; g__Erysipelotrichaceae_UCG-003; s__uncultured_bacterium_g__Erysipelotrichaceae_UCG-003	f__Erysipelotrichaceae
OTU704	Down	p__Firmicutes; c__Bacilli; o__Bacillales; f__Family_XI; g__Gemella; s__Gemella_sanguinis	f__Family_XI

OTU482	Down	p__Firmicutes; c__Clostridia; o__Clostridiales; f__Lachnospiraceae; g__[Eubacterium]_ventriosum_group; s__unclassified_g__[Eubacterium]_ventriosum_group	f__Lachnospiraceae
OTU477	Down	p__Firmicutes; c__Clostridia; o__Clostridiales; f__Lachnospiraceae; g__[Eubacterium]_ventriosum_group; s__uncultured_bacterium_g__[Eubacterium]_ventriosum_group	f__Lachnospiraceae
OTU279	Down	p__Firmicutes; c__Clostridia; o__Clostridiales; f__Lachnospiraceae; g__Blautia; s__unclassified_g__Blautia	f__Lachnospiraceae
OTU485	Down	p__Firmicutes; c__Clostridia; o__Clostridiales; f__Lachnospiraceae; g__Blautia; s__uncultured_Ruminococcus_sp._g__Blautia	f__Lachnospiraceae
OTU272	Down	p__Firmicutes; c__Clostridia; o__Clostridiales; f__Lachnospiraceae; g__Coprococcus_2; s__unclassified_g__Coprococcus_2	f__Lachnospiraceae
OTU254	Down	p__Firmicutes; c__Clostridia; o__Clostridiales; f__Lachnospiraceae; g__Dorea; s__uncultured_bacterium_g__Dorea	f__Lachnospiraceae
OTU129	Down	p__Firmicutes; c__Clostridia; o__Clostridiales; f__Lachnospiraceae; g__Fusicatenibacter; s__unclassified_g__Fusicatenibacter	f__Lachnospiraceae
OTU25	Down	p__Firmicutes; c__Clostridia; o__Clostridiales; f__Lachnospiraceae; g__Lachnoclostridium; s__Ruminococcus_torques_ATCC_27756	f__Lachnospiraceae
OTU259	Down	p__Firmicutes; c__Clostridia; o__Clostridiales; f__Lachnospiraceae; g__Lachnoclostridium; s__unclassified_g__Lachnoclostridium	f__Lachnospiraceae
OTU666	Down	p__Firmicutes; c__Clostridia; o__Clostridiales; f__Lachnospiraceae; g__Lachnoclostridium; s__unclassified_g__Lachnoclostridium	f__Lachnospiraceae
OTU353	Down	p__Firmicutes; c__Clostridia; o__Clostridiales; f__Lachnospiraceae; g__Lachnospiraceae_NK4A136_group; s__uncultured_organism_g__Lachnospiraceae_NK4A136_group	f__Lachnospiraceae
OTU853	Down	p__Firmicutes; c__Clostridia; o__Clostridiales; f__Lachnospiraceae; g__Lachnospiraceae_UCG-010; s__uncultured_organism_g__Lachnospiraceae_UCG-010	f__Lachnospiraceae
OTU111	Down	p__Firmicutes; c__Clostridia; o__Clostridiales; f__Lachnospiraceae; g__unclassified_f__Lachnospiraceae; s__unclassified_f__Lachnospiraceae	f__Lachnospiraceae
OTU337	Down	p__Firmicutes; c__Clostridia; o__Clostridiales; f__Lachnospiraceae; g__unclassified_f__Lachnospiraceae; s__unclassified_f__Lachnospiraceae	f__Lachnospiraceae
OTU623	Down	p__Firmicutes; c__Clostridia; o__Clostridiales; f__Lachnospiraceae; g__unclassified_f__Lachnospiraceae; s__unclassified_f__Lachnospiraceae	f__Lachnospiraceae

OTU629	Down	p__Firmicutes; c__Clostridia; o__Clostridiales; f__Lachnospiraceae; g__unclassified_f__Lachnospiraceae; s__unclassified_f__Lachnospiraceae	f__Lachnospiraceae
OTU170	Down	p__Proteobacteria; c__Alphaproteobacteria; o__Rhizobiales; f__Methylobacteriaceae; g__Methylobacterium; s__unclassified_g__Methylobacterium	f__Methylobacteriaceae
OTU341	Down	p__Tenericutes; c__Mollicutes; o__Mollicutes_RF9; f__norank_o__Mollicutes_RF9; g__norank_o__Mollicutes_RF9; s__uncultured_bacterium_g__norank_o__Mollicutes_RF9	f__norank
OTU147	Down	p__Saccharibacteria; c__norank_p__Saccharibacteria; o__norank_p__Saccharibacteria; f__norank_p__Saccharibacteria; g__norank_p__Saccharibacteria; s__Candidatus_Saccharibacteria_bacterium_UB2523	f__norank
OTU42	Down	p__Saccharibacteria; c__norank_p__Saccharibacteria; o__norank_p__Saccharibacteria; f__norank_p__Saccharibacteria; g__norank_p__Saccharibacteria; s__TM7_phylum_sp._oral_clone_DR034	f__norank
OTU41	Down	p__Saccharibacteria; c__norank_p__Saccharibacteria; o__norank_p__Saccharibacteria; f__norank_p__Saccharibacteria; g__norank_p__Saccharibacteria; s__TM7_phylum_sp._oral_clone_FR058	f__norank
OTU138	Down	p__Saccharibacteria; c__norank_p__Saccharibacteria; o__norank_p__Saccharibacteria; f__norank_p__Saccharibacteria; g__norank_p__Saccharibacteria; s__unclassified_g__norank_p__Saccharibacteria	f__norank
OTU367	Down	p__Saccharibacteria; c__norank_p__Saccharibacteria; o__norank_p__Saccharibacteria; f__norank_p__Saccharibacteria; g__norank_p__Saccharibacteria; s__unclassified_g__norank_p__Saccharibacteria	f__norank
OTU521	Down	p__Proteobacteria; c__Gammaproteobacteria; o__Pasteurellales; f__Pasteurellaceae; g__Haemophilus; s__Haemophilus_parainfluenzae_T3T1	f__Pasteurellaceae
OTU230	Down	p__Bacteroidetes; c__Bacteroidia; o__Bacteroidales; f__Porphyromonadaceae; g__Barnesiella; s__unclassified_g__Barnesiella	f__Porphyromonadaceae
OTU537	Down	p__Bacteroidetes; c__Bacteroidia; o__Bacteroidales; f__Porphyromonadaceae; g__Odoribacter; s__Odoribacter_splanchnicus_DSM_20712	f__Porphyromonadaceae
OTU677	Down	p__Bacteroidetes; c__Bacteroidia; o__Bacteroidales; f__Rikenellaceae; g__Alistipes; s__Alistipes_putredinis_DSM_17216	f__Rikenellaceae
OTU231	Down	p__Firmicutes; c__Clostridia; o__Clostridiales; f__Ruminococcaceae; g__[Eubacterium]_coprostanoligenes_group; s__uncultured_bacterium_g__[Eubacterium]_coprostanoligenes_group	f__Ruminococcaceae

OTU680	Down	p__Firmicutes; c__Clostridia; o__Clostridiales; f__Ruminococcaceae; g__[Eubacterium]_coprostanoligenes_group; s__uncultured_bacterium_g__[Eubacterium]_coprostanoligenes_group	f__Ruminococcaceae
OTU235	Down	p__Firmicutes; c__Clostridia; o__Clostridiales; f__Ruminococcaceae; g__Ruminiclostridium; s__uncultured_organism_g__Ruminiclostridium	f__Ruminococcaceae
OTU181	Down	p__Firmicutes; c__Clostridia; o__Clostridiales; f__Ruminococcaceae; g__Ruminococcaceae_UCG-005; s__uncultured_prokaryote_g__Ruminococcaceae_UCG-005	f__Ruminococcaceae
OTU789	Down	p__Firmicutes; c__Clostridia; o__Clostridiales; f__Ruminococcaceae; g__Ruminococcaceae_UCG-013; s__uncultured_organism_g__Ruminococcaceae_UCG-013	f__Ruminococcaceae
OTU276	Down	p__Firmicutes; c__Clostridia; o__Clostridiales; f__Ruminococcaceae; g__Ruminococcaceae_UCG-014; s__unclassified_g__Ruminococcaceae_UCG-014	f__Ruminococcaceae
OTU405	Down	p__Firmicutes; c__Clostridia; o__Clostridiales; f__Ruminococcaceae; g__Ruminococcaceae_UCG-014; s__uncultured_bacterium_g__Ruminococcaceae_UCG-014	f__Ruminococcaceae
OTU813	Down	p__Firmicutes; c__Clostridia; o__Clostridiales; f__Ruminococcaceae; g__Ruminococcaceae_UCG-014; s__uncultured_organism_g__Ruminococcaceae_UCG-014	f__Ruminococcaceae
OTU389	Down	p__Firmicutes; c__Clostridia; o__Clostridiales; f__Ruminococcaceae; g__Ruminococcaceae_UCG-014; s__uncultured_Ruminococcaceae_bacterium_g__Ruminococcaceae_UCG-014	f__Ruminococcaceae
OTU766	Down	p__Firmicutes; c__Clostridia; o__Clostridiales; f__Ruminococcaceae; g__Ruminococcus_1; s__Ruminococcus_bicirculans	f__Ruminococcaceae
OTU725	Down	p__Firmicutes; c__Clostridia; o__Clostridiales; f__Ruminococcaceae; g__Ruminococcus_2; s__uncultured_bacterium_g__Ruminococcus_2	f__Ruminococcaceae
OTU526	Down	p__Firmicutes; c__Clostridia; o__Clostridiales; f__Ruminococcaceae; g__uncultured_f__Ruminococcaceae; s__unclassified_g__uncultured_f__Ruminococcaceae	f__Ruminococcaceae
OTU834	Down	p__Firmicutes; c__Bacilli; o__Lactobacillales; f__Streptococcaceae; g__Streptococcus; s__Streptococcus_salivarius	f__Streptococcaceae
OTU395	Down	p__Firmicutes; c__Bacilli; o__Lactobacillales; f__Streptococcaceae; g__Streptococcus; s__unclassified_g__Streptococcus	f__Streptococcaceae
OTU191	Down	p__Firmicutes; c__Negativicutes; o__Selenomonadales; f__Veillonellaceae; g__Dialister; s__unclassified_g__Dialister	f__Veillonellaceae
OTU474	Up	p__Firmicutes; c__Negativicutes; o__Selenomonadales; f__Acidaminococcaceae; g__Acidaminococcus; s__uncultured_organism_g__Acidaminococcus	f__Acidaminococcaceae

OTU829	Up	p__Proteobacteria; c__Betaproteobacteria; o__Burkholderiales; f__Alcaligenaceae; g__Sutterella; s__Sutterella_wadsworthensis_2_1_59BFAA	f__Alcaligenaceae
OTU832	Up	p__Actinobacteria; c__Actinobacteria; o__Bifidobacteriales; f__Bifidobacteriaceae; g__Alloscardovia; s__Alloscardovia_omnicolens	f__Bifidobacteriaceae
OTU172	Up	p__Bacteroidetes; c__Bacteroidia; o__Bacteroidales; f__Bacteroidaceae; g__Bacteroides; s__Bacteroides_cellulosilyticus_DSM_14838	f__Bacteroidaceae
OTU849	Up	p__Bacteroidetes; c__Bacteroidia; o__Bacteroidales; f__Bacteroidaceae; g__Bacteroides; s__Bacteroides_thetaiotaomicron	f__Bacteroidaceae
OTU86	Up	p__Bacteroidetes; c__Bacteroidia; o__Bacteroidales; f__Bacteroidaceae; g__Bacteroides; s__unclassified_g__Bacteroides	f__Bacteroidaceae
OTU622	Up	p__Firmicutes; c__Clostridia; o__Clostridiales; f__Clostridiaceae_1; g__Sarcina; s__uncultured_Sarcina_sp._g__Sarcina	f__Clostridiaceae_1
OTU15	Up	p__Actinobacteria; c__Actinobacteria; o__Coriobacteriales; f__Coriobacteriaceae; g__Atopobium; s__unclassified_g__Atopobium	f__Coriobacteriaceae
OTU614	Up	p__Actinobacteria; c__Actinobacteria; o__Coriobacteriales; f__Coriobacteriaceae; g__uncultured_f__Coriobacteriaceae; s__metagenome	f__Coriobacteriaceae
OTU759	Up	p__Bacteroidetes; c__Flavobacteriia; o__Flavobacteriales; f__Flavobacteriaceae; g__Flavobacterium; s__Flavobacterium_anhuiense	f__Flavobacteriaceae
OTU780	Up	p__Fusobacteria; c__Fusobacteriia; o__Fusobacteriales; f__Fusobacteriaceae; g__Fusobacterium; s__unclassified_g__Fusobacterium	f__Fusobacteriaceae
OTU592	Up	p__Bacteroidetes; c__Bacteroidia; o__Bacteroidales; f__Prevotellaceae; g__Alloprevotella; s__uncultured_organism_g__Alloprevotella	f__Prevotellaceae
OTU455	Up	p__Bacteroidetes; c__Bacteroidia; o__Bacteroidales; f__Prevotellaceae; g__Prevotella_7; s__uncultured_organism_g__Prevotella_7	f__Prevotellaceae
OTU407	Up	p__Bacteroidetes; c__Bacteroidia; o__Bacteroidales; f__Prevotellaceae; g__Prevotella_9; s__uncultured_bacterium_g__Prevotella_9	f__Prevotellaceae
OTU662	Up	p__Bacteroidetes; c__Bacteroidia; o__Bacteroidales; f__Prevotellaceae; g__Prevotella_9; s__uncultured_bacterium_g__Prevotella_9	f__Prevotellaceae

OTU184	Up	p__Firmicutes; c__Clostridia; o__Clostridiales; f__Ruminococcaceae; g__[Eubacterium]_coprostanoligenes_group; s__human_gut_metagenome	f__Ruminococcaceae
OTU727	Up	p__Firmicutes; c__Clostridia; o__Clostridiales; f__Ruminococcaceae; g__Flavonifractor; s__Flavonifractor_plautii	f__Ruminococcaceae
OTU690	Up	p__Firmicutes; c__Negativicutes; o__Selenomonadales; f__Veillonellaceae; g__Dialister; s__human_gut_metagenome	f__Veillonellaceae
OTU639	Up	p__Firmicutes; c__Negativicutes; o__Selenomonadales; f__Veillonellaceae; g__Dialister; s__uncultured_bacterium_g__Dialister	f__Veillonellaceae
OTU457	Up	p__Firmicutes; c__Negativicutes; o__Selenomonadales; f__Veillonellaceae; g__Megasphaera; s__Megasphaera_elsdenii_DSM_20460	f__Veillonellaceae
OTU84	Up	p__Firmicutes; c__Negativicutes; o__Selenomonadales; f__Veillonellaceae; g__Megasphaera; s__unclassified_g__Megasphaera	f__Veillonellaceae
OTU778	Up	p__Firmicutes; c__Negativicutes; o__Selenomonadales; f__Veillonellaceae; g__Veillonella; s__unclassified_g__Veillonella	f__Veillonellaceae
OTU651	Up	p__Verrucomicrobia; c__Verrucomicrobiae; o__Verrucomicrobiales; f__Verrucomicrobiaceae; g__Akkermansia; s__unclassified_g__Akkermansia	f__Verrucomicrobiaceae

Table S2B. Discriminatory KEGG pathways between SCZ microbiota and HC microbiota recipient mice.

ID	Relative Abundance in 'SCZ microbiota' recipient mice		Relative Abundance in 'HC microbiota' recipient mice		Pathways	p-value	SCZ Relative to HCs
	Mean	Std. Deviation	Mean	Std. Deviation			
ko00564	220399.72	7284.57	200166.22	5447.65	Glycerophospholipid metabolism	<0.001	Up
ko00561	170929.70	13250.00	185647.36	8305.54	Glycerolipid metabolism	0.008	Down
ko00563	7382.06	441.30	6861.41	191.09	Glycosylphosphatidylinositol-anchor biosynthesis	0.004	Up
ko00604	92824.21	13225.25	111411.33	17201.08	Glycosphingolipid biosynthesis - ganglio series	0.007	Down
ko00603	147101.95	16954.69	185335.03	30526.68	Glycosphingolipid biosynthesis - globo series	<0.001	Down
ko04071	12543.24	743.87	10600.41	572.81	Sphingolipid signaling pathway	<0.001	Up
ko00600	342791.14	26563.22	435281.56	56818.85	Sphingolipid metabolism	<0.001	Down
ko00640	329542.18	7767.56	312127.54	6625.26	Propanoate metabolism	<0.001	Up
ko00592	9420.77	474.36	7604.62	628.47	alpha-Linolenic acid metabolism	<0.001	Up
ko00590	8083.28	778.74	10926.75	695.72	Arachidonic acid metabolism	<0.001	Down
ko01040	61474.97	1934.08	57080.00	3034.58	Biosynthesis of unsaturated fatty acids	0.001	Up
ko00970	712524.00	11758.88	694819.87	7467.11	Aminoacyl-tRNA biosynthesis	0.003	Up
ko01230	2080022.55	34503.69	1962968.41	16114.75	Biosynthesis of amino acids	<0.001	Up
ko00260	513174.46	7577.37	502186.82	3684.24	Glycine, serine and threonine metabolism	0.003	Up
ko00473	57038.11	862.00	54435.74	1039.97	D-Alanine metabolism	<0.001	Up
ko00300	273702.70	7146.57	254641.44	4048.78	Lysine biosynthesis	<0.001	Up
ko00290	245337.70	11741.55	223767.26	5693.26	Valine, leucine and isoleucine biosynthesis	<0.001	Up
ko00400	304598.09	4849.41	294549.61	3474.69	Phenylalanine, tyrosine and tryptophan biosynthesis	<0.001	Up
ko00270	478365.49	9230.89	458132.12	6348.86	Cysteine and methionine metabolism	<0.001	Up
ko04973	15360.08	938.70	20765.37	2065.06	Carbohydrate digestion and absorption	<0.001	Down

ko01200	1680912.20	19731.15	1612884.13	8962.38	Carbon metabolism	<0.001	Up
ko00620	686439.86	12873.43	653416.97	5426.69	Pyruvate metabolism	<0.001	Up
ko00010	595850.51	16902.40	577452.12	12723.27	Glycolysis / Gluconeogenesis	0.025	Up
ko00533	711.81	190.72	369.63	92.14	Glycosaminoglycan biosynthesis - keratan sulfate	<0.001	Up
ko00532	962.08	143.87	647.39	84.36	Glycosaminoglycan biosynthesis - chondroitin sulfate	<0.001	Down
ko00534	1396.86	63.17	1621.45	144.78	Glycosaminoglycan biosynthesis - heparan sulfate	<0.001	Down
ko00730	176996.24	4692.57	163795.89	5826.54	Thiamine metabolism	<0.001	Up
ko00230	1260655.76	15866.35	1220764.51	20427.46	Purine metabolism	<0.001	Up
ko00240	989324.92	14125.23	961965.92	21673.52	Pyrimidine metabolism	0.009	Up
ko00650	269911.08	13424.28	248023.42	4405.37	Butanoate metabolism	<0.001	Up
ko00630	542469.62	7986.26	523413.64	8051.18	Glyoxylate and dicarboxylate metabolism	<0.001	Up
ko00440	47717.46	2828.06	29336.36	1843.18	Phosphonate and phosphinate metabolism	<0.001	Up
ko00680	428098.59	7692.01	404834.85	10221.88	Methane metabolism	<0.001	Up

Table S3A. Differential fecal metabolites between SCZ microbiota and HC microbiota recipient mice.

Platform	Metabolites	Relative Abundance in 'SCZ microbiota' recipient mice		Relative Abundance in 'HC microbiota' recipient mice		SCZ/ CON	KEGG pathway
LC	PE(22:4(7Z,10Z,13Z,16Z)/20:4(5Z,8Z,11Z,14Z))	1132.21	495.28	301.61	177.26	Up	Lipids metabolism
LC	PE(22:6(4Z,7Z,10Z,13Z,16Z,19Z)/20:2(11Z,14Z))	402.43	159.71	74.18	61.72	Up	Lipids metabolism
LC	PC(22:6(4Z,7Z,10Z,13Z,16Z,19Z)/18:2(9Z,12Z))	3739.94	1799.172	1440.87	630.28	Up	Lipids metabolism
LC	LysoPC(P-18:0)	941.64	130.96	243.99	84.74	Up	Lipids metabolism
LC	LysoPC(22:4(7Z,10Z,13Z,16Z))	440.46	122.44	32.44	60.86	Up	Lipids metabolism
LC	LysoPC(P-18:1(9Z))	480.86	258.64	10.70	28.99	Up	Lipids metabolism
LC	LysoPE(22:0/0:0)	486.21	116.85	141.17	25.71	Up	Lipids metabolism
LC	DG(18:2n6/0:0/20:4n3)	5616.97	2164.84	2563.66	714.76	Up	Lipids metabolism
LC	DG(18:2n6/0:0/20:4n6)	3527.33	1203.07	1458.31	520.27	Up	Lipids metabolism
GC	glycerol	372.40	217.69	56.46	88.11	Up	Lipids metabolism
NMR	Glutamine	0.0805	0.00315	0.0890	0.00252	Down	Amino acid metabolism
NMR	Glutamate	1.4775	0.06283	1.5359	0.08078	Down	Amino acid metabolism
NMR	Isobutyrate	0.0491	0.00484	0.0499	0.00570	Down	Amino acid metabolism
NMR	Aspartate	0.0670	0.00216	0.0611	0.00291	Up	Amino acid metabolism
NMR	Histidine	0.0077	0.00102	0.0068	0.00068	Up	Amino acid metabolism
GC	glycine	42.34	14.89	29.15	7.31	Up	Amino acid metabolism
NMR	Leucine	1.8411	0.12622	1.1464	0.12283	Up	Amino acid metabolism
GC	D-alanyl-D-alanine	28.95	10.54	16.38	7.65	Up	Amino acid metabolism

GC	alanine	201.79	92.95	101.66	31.02	Up	Amino acid metabolism
LC	N-Undecanoylglycine	753.54	379.85	311.44	119.76	Up	Amino acid metabolism
GC	serine	55.34	26.15	22.35	7.77	Up	Amino acid metabolism
GC	valine	74.16	35.82	26.86	10.16	Up	Amino acid metabolism
GC	ornithine	70.41	25.37	23.09	23.01	Up	Amino acid metabolism
GC	phenylalanine	38.09	14.44	12.41	7.77	Up	Amino acid metabolism
GC	tyrosine	175.51	96.66	53.41	38.79	Up	Amino acid metabolism
GC	asparagine	17.13	4.83	5.12	6.25	Up	Amino acid metabolism
GC	Isoleucine	48.28	23.87	13.38	8.05	Up	Amino acid metabolism
GC	lysine	513.52	243.71	137.76	190.34	Up	Amino acid metabolism
LC	L-Arginine	957.02	151.88	217.49	117.29	Up	Amino acid metabolism
LC	N-Arachidonoyl glycine	1131.20	227.15	196.20	121.86	Up	Amino acid metabolism
LC	Tetrahydrodeoxycorticosterone	1.81	2.22	530.15	421.22	Down	Others
LC	17-HDoHE	2.47	3.84	500.50	442.63	Down	Others
LC	D-4'-Phosphopantothenate	46.63	126.00	1022.42	944.51	Down	Others
LC	6-Hydroxypentadecanedioic acid	63.88	21.02	981.60	626.88	Down	Others
LC	15beta-Hydroxydesogestrel	37.45	14.37	512.78	459.85	Down	Others
LC	2-Hydroxyenterodiol	361.58	137.81	1212.12	411.32	Down	Others
LC	4-O-beta-D-Glucopyranuronosyl-L-fucose	406.99	112.31	1184.30	463.46	Down	Others
LC	1,11-Undecanedicarboxylic acid	74.13	54.00	395.53	156.93	Down	Others
LC	8-Hydroxyoctanoate	339.38	115.85	703.28	202.32	Down	Others
NMR	Uracil	0.0106	0.00512	0.0200	0.00673	Down	Others
GC	Galactinol	932.84	418.18	1684.60	324.59	Down	Others
GC	beta-Mannosylglycerate	437.75	207.93	676.28	245.14	Down	Others

GC	Glucose-1-phosphate	56.20	19.07	81.71	23.31	Down	Others
NMR	α-Glucose	0.1501	0.01558	0.2146	0.04182	Down	Others
NMR	Inosine	0.0200	0.00532	0.0283	0.00603	Down	Others
NMR	Formate	0.0042	0.00398	0.0059	0.00415	Down	Others
NMR	Nicotinate	0.0082	0.00237	0.0115	0.00290	Down	Others
GC	D-(glycerol-phosphate)	48.89	16.03	66.12	12.89	Down	Others
NMR	Adenosine	0.0200	0.00452	0.0263	0.00557	Down	Others
NMR	Acetate	4.4102	0.49249	5.4129	1.07281	Down	Others
NMR	Fumarate	0.0016	0.00088	0.0019	0.00054	Down	Others
NMR	β-Glucose	0.1991	0.01736	0.2352	0.03904	Down	Others
NMR	Allantoin	0.1189	0.01702	0.1337	0.02890	Down	Others
NMR	myo-Inositol	0.1391	0.00421	0.1562	0.00638	Down	Others
NMR	Butyrate	1.9641	0.04489	2.1785	0.15440	Down	Others
NMR	Dimethylglycine	0.0635	0.00356	0.0689	0.00938	Down	Others
NMR	Choline	0.0919	0.00377	0.0992	0.00451	Down	Others
NMR	Betaine	0.2224	0.02562	0.2361	0.01328	Down	Others
NMR	Malate	0.0932	0.00400	0.0979	0.00493	Down	Others
NMR	α-Ketobutyrate	1.7544	0.20400	1.8290	0.31877	Down	Others
NMR	Trimethylamine	0.0567	0.00123	0.0590	0.00297	Down	Others
NMR	Malonate	0.0761	0.00262	0.0789	0.00260	Down	Others
NMR	β-Galactose	0.0503	0.00297	0.0519	0.00258	Down	Others
NMR	Glycolic acid	0.1126	0.00539	0.1156	0.00570	Down	Others
NMR	Pyroglutamate	0.0741	0.00296	0.0750	0.00303	Down	Others
NMR	Acetoacetate	0.6924	0.04863	0.6976	0.04746	Down	Others
NMR	Dimethylamine	0.0667	0.00309	0.0654	0.00628	Up	Others

NMR	Bile acid	0.0318	0.00769	0.0305	0.00548	Up	Others
NMR	3-Methylxanthine	0.0035	0.00083	0.0033	0.00066	Up	Others
NMR	3-Hydroxybutyrate	0.6800	0.03344	0.6379	0.05421	Up	Others
NMR	Methylamine	0.0706	0.00337	0.0660	0.00329	Up	Others
NMR	Succinate	0.9199	0.34322	0.8499	0.18471	Up	Others
NMR	Uridine	0.0069	0.00062	0.0064	0.00067	Up	Others
NMR	Formylglycine	0.0037	0.00106	0.0033	0.00080	Up	Others
NMR	Aminohippurate	0.0291	0.00184	0.0239	0.00157	Up	Others
NMR	1-Methylhistidine	0.0869	0.02668	0.0610	0.01911	Up	Others
GC	Ethanolamine	44.25	17.17	27.23	12.69	Up	Others
LC	N1-Acetylspermidine	1001.41	542.78	492.52	120.58	Up	Others
LC	3-Hydroxyhexadecanoylcarnitine	1261.88	506.61	620.49	441.15	Up	Others
LC	1- Heptadecanoylglycerophosphoethanolamine	25325.69	6246.94	12271.03	2782.24	Up	Others
LC	12-Keto-tetrahydro-leukotriene B4	1092.96	401.80	505.12	148.09	Up	Others
LC	(4-Hydroxybenzoyl)choline	3630.60	828.29	1591.97	735.41	Up	Others
LC	3-Polyprenyl-4,5-dihydroxybenzoate	1188.53	244.23	500.77	93.70	Up	Others
LC	Pregnanetriol	853.44	83.09	357.86	55.93	Up	Others
LC	3a,7a-Dihydroxycoprostanic acid	856.26	108.10	355.12	90.54	Up	Others
LC	Brevifolincarboxylic acid 9-sulfate	1198.63	393.95	493.13	562.93	Up	Others
LC	Glycocholic acid	1749.86	227.59	715.68	157.20	Up	Others
LC	2-Oleoylglycerophosphocholine	1867.56	925.05	747.63	111.89	Up	Others
LC	2-Phenylacetamide	754.82	297.14	286.83	78.13	Up	Others
LC	11beta,20-Dihydroxy-3-oxopregn-4-en-21-oic acid	944.38	172.29	357.34	72.93	Up	Others

LC	Vitamin A2 aldehyde	4145.90	1558.51	1544.94	562.05	Up	Others
LC	Estrone	889.14	141.00	324.04	55.37	Up	Others
GC	Methyl-beta-D-galactopyranoside	17.04	4.16	6.20	5.17	Up	Others
LC	5-Methoxydimethyltryptamine	630.36	129.21	209.86	61.20	Up	Others
LC	Leukotriene E4	1600.59	673.86	494.13	42.26	Up	Others
LC	Lithocholyltaurine	683.12	127.13	225.64	62.87	Up	Others
GC	citrulline	15.09	3.66	4.97	3.63	Up	Others
LC	p-Hydroxynonanophenone	1603.98	880.43	499.69	92.15	Up	Others
LC	Vitamin A2	4409.21	1701.09	1307.92	835.82	Up	Others
LC	Adrenosterone	1179.60	243.80	340.40	55.00	Up	Others
LC	13'-Hydroxy-alpha-tocotrienol	1904.29	1061.42	539.34	323.05	Up	Others
LC	3-Oxotetradecanoic acid glycerides	753.45	187.21	206.31	71.01	Up	Others
LC	Cortisone acetate	1399.46	241.18	383.12	123.84	Up	Others
LC	6,8-Dihydroxypurine	605.98	139.77	161.48	55.36	Up	Others
LC	16a-Hydroxyestrone	424.47	89.87	105.74	24.34	Up	Others
LC	Deoxycholic acid 3-glucuronide	753.48	412.12	176.33	36.45	Up	Others
LC	2-Phenylethyl acetate	492.34	228.16	110.42	92.79	Up	Others
LC	All-trans-hexaprenyl diphosphate	1103.81	157.49	246.16	127.28	Up	Others
LC	Pyridoxamine	975.50	185.57	210.93	79.63	Up	Others
LC	9'-Carboxy-gamma-tocotrienol	1436.41	306.63	308.78	166.10	Up	Others
LC	Homocysteine thiolactone	501.84	87.11	105.30	72.67	Up	Others
LC	S-Methyl benzenecarbothioate	892.16	160.32	186.91	80.71	Up	Others
LC	17a-Ethynylestradiol	559.97	162.05	111.22	48.52	Up	Others
LC	3-Oxododecanoic acid glycerides	728.95	212.87	144.61	74.20	Up	Others
LC	7-Hydroxyterpineol 8-glucoside	1487.84	411.03	268.49	99.55	Up	Others

LC	12a-Hydroxy-3-oxocholadienic acid	555.04	99.25	92.93	49.27	Up	Others
LC	3-Oxo-4,6-choladienoic acid	631.38	155.03	90.31	127.24	Up	Others
LC	gamma-Chaconine	462.57	178.53	65.74	39.32	Up	Others
LC	Tauroursocholic acid	752.05	155.42	84.39	31.89	Up	Others
LC	LysoPE(22:4(7Z,10Z,13Z,16Z)/0:0)	373.82	83.86	40.84	32.07	Up	Others
LC	2-Phenylethyl pentanoate	1157.03	199.66	119.06	137.92	Up	Others
LC	2-trans,4-cis-Decadienoylcarnitine	1351.94	322.32	138.49	106.09	Up	Others
LC	19-Hydroxydeoxycorticosterone	856.24	239.69	78.82	64.17	Up	Others
LC	2-sulfamoylacetylphenol	900.38	406.28	71.76	67.13	Up	Others
LC	11'-Carboxy-alpha-tocotrienol	483.69	135.62	36.28	43.14	Up	Others
LC	3,4-Dimethyl-5-pentyl-2-furanundecanoic acid	875.39	181.24	56.60	58.65	Up	Others
LC	Etamiphylline	668.25	337.04	40.96	15.50	Up	Others
LC	11'-Carboxy-gamma-tocotrienol	430.56	104.05	24.65	33.92	Up	Others
LC	13'-Carboxy-gamma-tocotrienol	369.80	101.60	14.14	10.02	Up	Others
LC	Chenodeoxyglycocholic acid	732.16	99.09	25.87	37.47	Up	Others
LC	2-Heptenoic acid	1548.97	1132.34	45.26	11.43	Up	Others
LC	Cholesterol glucuronide	1528.72	287.59	44.45	11.34	Up	Others
LC	2-hydroxydesipramine	568.38	425.70	16.13	11.08	Up	Others
LC	Octaprenyl diphosphate	1287.30	435.12	33.19	68.02	Up	Others
LC	2-Hexaprenyl-6-methoxyphenol	454.43	344.61	7.41	13.56	Up	Others
LC	12-oxo-20-dihydroxy-leukotriene B4	2174.83	538.37	33.71	52.29	Up	Others
LC	3a,21-Dihydroxy-5b-pregnane-11,20-dione	763.53	295.51	11.60	17.77	Up	Others
LC	(S)-9-Hydroxy-10-undecenoic acid	386.86	179.80	5.39	9.41	Up	Others

LC	7a,12a-Dihydroxy-3-oxo-4-cholenoic acid	340.99	103.12	4.10	8.24	Up	Others
LC	3-Hexaprenyl-4-hydroxy-5-methoxybenzoic acid	449.48	162.17	0.37	0.46	Up	Others

Table S3B. Differential serum metabolites between SCZ microbiota and HC microbiota recipient mice.

Platform	Metabolites	Relative Abundance in 'SCZ microbiota' recipient mice		Relative Abundance in 'HC microbiota' recipient mice		SCZ/HC	KEGG pathway
GC	glutamine	1.86	0.50	1.21	0.37	Up	Amino acid metabolism
GC	alanine	3.39	1.20	2.32	0.83	Up	Amino acid metabolism
GC	asparagine	1.51	0.63	0.85	0.17	Up	Amino acid metabolism
GC	beta-Alanine	3.39	1.20	2.32	0.83	Up	Amino acid metabolism
GC	glycine	1.08	0.58	0.57	0.13	Up	Amino acid metabolism
GC	leucine	1.41	0.77	0.66	0.49	Up	Amino acid metabolism
GC	lysine	6.87	2.42	4.48	1.27	Up	Amino acid metabolism
GC	methionine	3.20	1.24	2.13	0.50	Up	Amino acid metabolism
GC	ornithine	0.84	0.34	0.45	0.11	Up	Amino acid metabolism
GC	phenylalanine	3.24	1.40	2.25	0.49	Up	Amino acid metabolism
GC	proline	2.72	0.72	1.97	0.55	Up	Amino acid metabolism
LC	S-Adenosylhomocysteine	3.13	2.34	7.14	3.43	Down	Amino acid metabolism
GC	serine	1.56	0.50	1.09	0.28	Up	Amino acid metabolism
GC	threonine	1.27	0.48	0.86	0.21	Up	Amino acid metabolism
LC	PE(18:2(9Z,12Z)/24:1(15Z))	27.15	46.60	195.54	134.53	Down	Lipids metabolism
LC	PE(20:0/P-18:1(11Z))	362.03	145.41	785.31	301.29	Down	Lipids metabolism
LC	PE(22:0/16:1(9Z))	290.19	76.54	496.56	144.74	Down	Lipids metabolism
LC	PE(22:1(13Z)/P-18:1(11Z))	81.81	46.06	253.89	133.60	Down	Lipids metabolism
LC	PE(22:2(13Z,16Z)/P-18:0)	4.73	8.02	50.80	39.17	Down	Lipids metabolism
LC	PE(22:5(7Z,10Z,13Z,16Z,19Z)/18:0)	6.70	7.59	18.48	6.13	Down	Lipids metabolism
LC	PE(P-16:0e/16:0)	8.65	10.42	57.53	47.82	Down	Lipids metabolism

LC	PE(P-18:1(9Z)/20:0)	286.55	86.27	513.65	161.61	Down	Lipids metabolism
LC	PE(P-18:1(9Z)/22:2(13Z,16Z))	38.66	19.92	105.41	47.06	Down	Lipids metabolism
LC	PC(18:2(9Z,12Z)/16:1(9Z))	333.18	268.50	1070.72	425.36	Down	Lipids metabolism
LC	PC(18:2(9Z,12Z)/18:3(6Z,9Z,12Z))	5.26	5.42	28.50	15.45	Down	Lipids metabolism
LC	PC(20:1(11Z)/22:6(4Z,7Z,10Z,13Z,16Z,19Z))	113.17	31.65	204.09	66.51	Down	Lipids metabolism
LC	PC(20:3(8Z,11Z,14Z)/22:6(4Z,7Z,10Z,13Z,16Z,19Z))	10.12	10.61	28.53	14.69	Down	Lipids metabolism
LC	PC(20:4(8Z,11Z,14Z,17Z)/20:4(5Z,8Z,11Z,14Z))	9.78	11.55	78.06	30.39	Down	Lipids metabolism
LC	PC(20:5(5Z,8Z,11Z,14Z,17Z)/P-18:1(11Z))	90.59	72.31	265.73	133.17	Down	Lipids metabolism
LC	PC(22:2(13Z,16Z)/16:1(9Z))	48.97	31.65	203.24	106.79	Down	Lipids metabolism
LC	PC(22:4(7Z,10Z,13Z,16Z)/16:0)	90.40	41.22	216.79	86.55	Down	Lipids metabolism
LC	PC(22:5(4Z,7Z,10Z,13Z,16Z)/16:1(9Z))	93.76	101.31	576.60	362.69	Down	Lipids metabolism
LC	PC(22:5(4Z,7Z,10Z,13Z,16Z)/22:6(4Z,7Z,10Z,13Z,16Z,19Z))	7.77	9.83	20.05	8.24	Down	Lipids metabolism
LC	PC(22:5(7Z,10Z,13Z,16Z,19Z)/16:0)	20.25	24.06	104.44	62.49	Down	Lipids metabolism
LC	PC(22:5(7Z,10Z,13Z,16Z,19Z)/18:0)	4.79	6.35	67.77	48.07	Down	Lipids metabolism
LC	PC(22:5(7Z,10Z,13Z,16Z,19Z)/P-18:1(11Z))	39.08	35.37	275.23	166.58	Down	Lipids metabolism
LC	PC(22:5(7Z,10Z,13Z,16Z,19Z)/P-18:1(9Z))	63.24	73.56	484.64	319.85	Down	Lipids metabolism
LC	PC(22:6(4Z,7Z,10Z,13Z,16Z,19Z)/16:0)	17.59	18.17	65.62	34.15	Down	Lipids metabolism
LC	PC(22:6(4Z,7Z,10Z,13Z,16Z,19Z)/18:1(9Z))	77.25	38.63	307.35	149.27	Down	Lipids metabolism

LC	PC(MonoMe(9,5)/MonoMe(9,5))	17.99	9.34	8.66	2.99	Up	Lipids metabolism
LC	PC(o-18:0/20:4(8Z,11Z,14Z,17Z))	17.39	21.39	92.05	56.64	Down	Lipids metabolism
LC	PC(P-18:0/22:4(7Z,10Z,13Z,16Z))	9.38	16.30	107.54	84.98	Down	Lipids metabolism
LC	PC(P-18:0/22:5(7Z,10Z,13Z,16Z,19Z))	52.93	56.66	244.40	128.50	Down	Lipids metabolism
LC	PC(P-18:1(11Z)/20:1(11Z))	144.58	173.02	796.16	477.29	Down	Lipids metabolism
LC	PC(P-18:1(11Z)/20:3(8Z,11Z,14Z))	9.97	11.58	57.53	30.47	Down	Lipids metabolism
LC	PC(P-18:1(11Z)/20:5(5Z,8Z,11Z, 14Z,17Z))	27.69	31.34	177.01	110.91	Down	Lipids metabolism
LC	PC(P-18:1(9Z)/20:4(5Z,8Z,11Z,14Z))	21.59	33.43	206.43	150.36	Down	Lipids metabolism
LC	PC(P-18:1(9Z)/20:4(8Z,11Z,14Z,17Z))	4.81	7.74	50.35	36.71	Down	Lipids metabolism
LC	PS(14:0/14:1(9Z))	49.81	31.53	211.14	138.07	Down	Lipids metabolism
LC	PS(14:1(9Z)/14:1(9Z))	2.66	4.38	20.20	15.25	Down	Lipids metabolism
LC	PS(18:0/18:2(9Z,12Z))	12.12	12.46	76.17	55.06	Down	Lipids metabolism
LC	PS(18:0/20:3(8Z,11Z,14Z))	66.79	78.93	429.80	334.31	Down	Lipids metabolism
LC	PS(18:0/22:5(7Z,10Z,13Z,16Z,19Z))	531.31	165.52	1026.63	326.66	Down	Lipids metabolism
LC	PS(18:1(9Z)/18:0)	155.05	136.50	702.62	504.88	Down	Lipids metabolism
LC	PS(22:6(4Z,7Z,10Z,13Z,16Z,19Z)/18:1(9Z))	21.17	15.29	40.20	10.26	Down	Lipids metabolism
LC	PI(20:4(5Z,8Z,11Z,14Z)/16:0)	229.60	135.12	844.26	583.20	Down	Lipids metabolism
LC	PI(22:6(4Z,7Z,10Z,13Z,16Z,19Z)/18:0)	15.08	14.97	47.89	25.32	Down	Lipids metabolism
LC	DG(15:0/22:0/0:0)	70.16	75.26	158.06	28.70	Down	Lipids metabolism
LC	DG(22:4(7Z,10Z,13Z,16Z)/22:5(4Z,7Z,10Z,13Z,16Z)/0:0)	54.80	76.12	167.50	76.33	Down	Lipids metabolism
LC	Glucosylceramide (d18:1/16:0)	3.83	2.51	6.59	1.40	Down	Lipids metabolism
LC	Glycerol tripropanoate	9.00	6.09	3.07	2.14	Up	Lipids metabolism

LC	LysoPC(24:0)	2.77	3.90	34.30	31.37	Down	Lipids metabolism
LC	lysoPC(26:0)	28.80	26.33	142.11	96.10	Down	Lipids metabolism
LC	PGP(18:1(9Z)/22:5(7Z,10Z,13Z,16Z,19Z))	40.28	12.72	26.38	6.49	Up	Lipids metabolism
LC	TG(15:0/14:1(9Z)/18:4(6Z,9Z,12Z,15Z))	123.44	39.75	196.24	45.40	Down	Lipids metabolism
LC	12-hydroxyheptadecanoic acid	36.79	17.42	55.42	8.06	Down	Others
LC	12-Hydroxystearic acid	5.27	3.85	12.62	5.63	Down	Others
LC	13'-Hydroxy-gamma-tocotrienol	2.91	2.83	7.56	3.58	Down	Others
GC	1-Methylhydantoin	21.22	5.81	15.01	3.57	Up	Others
LC	1-O-Sinapoylglucose	3.38	2.73	6.75	1.99	Down	Others
LC	1-Phenyl-1,3-heptadecanedione	333.16	284.30	768.45	143.43	Down	Others
LC	1-Stearoylglycerophosphoserine	38.74	12.26	60.05	14.20	Down	Others
LC	25-Hydroxyvitamin D3-26,23-lactone	24.19	18.49	125.00	85.51	Down	Others
GC	2-Amino-2-norbornanecarboxylic acid	0.01	0.01	0.03	0.01	Down	Others
GC	2-hydroxypyridine	0.17	0.03	0.13	0.04	Up	Others
LC	2-Polyprenyl-6-methoxyphenol	313.87	170.76	126.80	57.99	Up	Others
LC	2-Undecenal	2.77	2.83	7.08	3.06	Down	Others
LC	3, 5-Tetradecadiencarnitine	328.40	263.66	592.67	100.85	Down	Others
LC	3a,6b,7b-Trihydroxy-5b-cholanoic acid	237.80	206.78	732.07	196.67	Down	Others
LC	3b-Hydroxy-5-cholenoic acid	2505.72	1968.91	6232.45	1327.79	Down	Others
LC	3-Furanmethanol glucoside	43.63	26.85	79.22	14.79	Down	Others
LC	3-hydroxyhexadecanoic acid	3.93	2.74	6.82	1.59	Down	Others
LC	3-hydroxynonanoyl carnitine	35.08	13.53	21.68	5.60	Up	Others
LC	3-hydroxyundecanoyl carnitine	23.60	11.65	11.84	4.57	Up	Others
LC	3-Methyluridine	1.22	1.12	2.84	1.35	Down	Others

LC	4,6-Nonadecanedione	25.82	11.66	39.64	6.10	Down	Others
LC	4-Hydroxybenzaldehyde	0.87	0.82	2.37	0.86	Down	Others
LC	4-Hydroxydiphenylamine	4.52	2.62	7.99	1.86	Down	Others
LC	4-Hydroxytamoxifen-N-glucuronide	2.48	1.91	0.63	0.50	Up	Others
LC	5,6-Dihydroxyprostaglandin F1a	1.59	2.11	5.31	2.47	Down	Others
GC	5-Aminovaleric acid	0.45	0.24	0.20	0.10	Up	Others
LC	5-Hydroxy-7-(4-hydroxy-3-methoxyphenyl)-1-phenyl-3-heptanone	2.56	2.08	6.46	2.02	Down	Others
LC	6-Deoxohomodolichosterone	3.39	5.66	12.13	5.36	Down	Others
LC	7-Dehydrodesmosterol	7.62	10.40	32.67	16.66	Down	Others
LC	7-Ketocholesterol	137.67	121.80	483.47	220.54	Down	Others
LC	9,10-Epoxyoctadecenoic acid	0.94	1.36	13.51	6.80	Down	Others
LC	9-Oxooctadecanoic acid	2.54	4.41	9.61	5.97	Down	Others
GC	Adipamide	0.29	0.16	0.15	0.09	Up	Others
LC	alpha-Tocopherolquinone	7.74	1.36	5.07	2.06	Up	Others
GC	ascorbate	0.21	0.16	0.07	0.06	Up	Others
GC	Benzyl thiocyanate	1.31	0.49	0.78	0.37	Up	Others
GC	beta-Mannosylglycerate	9.66	2.65	7.28	1.29	Up	Others
LC	Bis-gamma-glutamylcysteinylbis-beta-alanine	40.47	33.40	4.05	5.24	Up	Others
LC	CerP(d18:1/16:0)	12.34	10.37	41.54	21.26	Down	Others
LC	Cholesta-4,6-dien-3-one	16.35	23.82	77.26	47.01	Down	Others
LC	Cholesteryl acetate	516.06	177.26	830.03	241.66	Down	Others
GC	creatine degr	0.40	0.16	0.26	0.05	Up	Others
GC	Cumic Acid	1.25	0.43	0.88	0.33	Up	Others

GC	D-erythro-sphingosine	0.53	0.09	0.44	0.08	Up	Others
GC	Dioctyl phthalate	0.39	0.12	0.28	0.04	Up	Others
LC	Dodecanoylcarnitine	61.32	63.92	179.22	64.00	Down	Others
GC	D-Talose	0.25	0.09	0.17	0.07	Up	Others
LC	Egonol glucoside	58.34	32.90	91.44	12.55	Down	Others
GC	Erythrose	0.22	0.07	0.15	0.05	Up	Others
GC	fumaric acid	0.46	0.19	0.24	0.06	Up	Others
GC	Galactonic acid	0.24	0.06	0.19	0.03	Up	Others
LC	gamma-Glutamyl-S-(1-propenyl)cysteine sulfoxide	0.70	0.65	1.67	0.57	Down	Others
LC	Heptadecanoic acid	9.11	5.77	15.15	2.39	Down	Others
LC	Homoanserine	1.64	1.67	3.95	1.43	Down	Others
LC	L-2-Amino-3-methylenehexanoic acid	183.33	33.05	106.75	25.12	Up	Others
LC	Lactosylceramide (d18:1/12:0)	6.14	7.99	53.11	40.25	Down	Others
GC	Leucrose	0.26	0.21	0.10	0.09	Up	Others
LC	Lithocholate 3-O-glucuronide	1.97	1.59	4.35	1.41	Down	Others
GC	L-Malic acid	0.50	0.29	0.24	0.10	Up	Others
GC	Maleimide	0.40	0.14	0.23	0.05	Up	Others
GC	malonic acid	5.89	3.17	8.54	1.55	Down	Others
GC	Mevalonic acid lactone	10.63	2.92	8.46	1.18	Up	Others
GC	oxalic acid	12.54	3.60	9.76	1.47	Up	Others
GC	oxoproline	31.29	12.36	22.46	2.97	Up	Others
GC	Pelargonic acid	0.57	0.16	0.45	0.05	Up	Others
LC	Pregnanetriol	59.67	23.14	106.14	41.55	Down	Others
GC	Sedoheptulose	1.78	0.56	2.41	0.58	Down	Others

GC	succinic acid	5.35	3.32	2.42	0.48	Up	Others
LC	Tetracosatetraenoic acid (24:4n-6)	30.07	12.95	46.27	10.01	Down	Others
GC	uracil	0.73	0.49	0.38	0.09	Up	Others
GC	urea	448.07	136.91	349.45	43.47	Up	Others

Table S3C. Differential hippocampus metabolites between SCZ microbiota and HC microbiota recipient mice.

Platform	Metabolites	Relative Abundance in 'SCZ microbiota' recipient mice		Relative Abundance in 'HC microbiota' recipient mice		SCZ/HC	KEGG pathway
GC	glutamic acid	172.18	13.62	192.49	26.65	Down	Amino acid metabolism
GC	Glutamine	0.65	0.08	0.56	0.07	Up	Amino acid metabolism
GC	asparagine	0.51	0.22	0.31	0.04	Up	Amino acid metabolism
GC	aspartic acid	182.34	37.72	210.21	13.05	Down	Amino acid metabolism
GC	beta-Alanine	0.21	0.04	0.14	0.03	Down	Amino acid metabolism
GC	D-alanyl-D-alanine	0.40	0.12	0.67	0.15	Down	Amino acid metabolism
LC	Glutathione	2568.81	858.44	1772.78	676.83	Up	Amino acid metabolism
GC	N-Acetyl-L-aspartic acid	4.66	0.30	4.31	0.28	Up	Amino acid metabolism
LC	Oxidized glutathione	2976.95	1085.08	4880.24	1505.56	Down	Amino acid metabolism
GC	proline	4.09	0.19	3.68	0.57	Up	Amino acid metabolism
GC	taurine	3.51	0.97	4.83	0.69	Down	Amino acid metabolism
GC	threonine	0.36	0.08	0.20	0.15	Up	Amino acid metabolism
LC	DG(22:6(4Z,7Z,10Z,13Z,16Z,19Z)/20:4(5Z,8Z,11Z,14Z)/0:0)	549.40	473.61	1324.64	700.69	Down	Lipids metabolism
LC	Glucosylceramide (d18:1/16:0)	595.37	172.71	825.15	283.53	Down	Lipids metabolism
LC	LysoPC(18:1(9Z))	62397.31	9621.14	70547.15	6097.75	Down	Lipids metabolism
LC	LysoPC(18:2(9Z,12Z))	2169.38	263.77	2462.55	230.19	Down	Lipids metabolism
LC	LysoPC(20:3(8Z,11Z,14Z))	964.70	274.36	1318.52	228.87	Down	Lipids metabolism
LC	LysoPC(20:4(5Z,8Z,11Z,14Z))	19688.35	5478.30	26503.15	4433.16	Down	Lipids metabolism
LC	LysoPC(22:4(7Z,10Z,13Z,16Z))	1126.95	252.12	1482.07	209.12	Down	Lipids metabolism
LC	LysoPC(22:5(7Z,10Z,13Z,16Z,19Z))	648.38	162.79	823.26	139.85	Down	Lipids metabolism

LC	LysoPC(22:6(4Z,7Z,10Z,13Z,16Z,19Z))	12399.78	2977.05	15358.76	2442.17	Down	Lipids metabolism
LC	LysoPE(20:4(8Z,11Z,14Z,17Z)/0:0)	13334.00	2699.71	16257.19	2791.53	Down	Lipids metabolism
LC	LysoPE(22:4(7Z,10Z,13Z,16Z)/0:0)	2633.38	478.02	3265.41	480.13	Down	Lipids metabolism
LC	LysoPE(22:6(4Z,7Z,10Z,13Z,16Z,19Z)/0:0)	11336.50	1705.24	9025.47	2838.69	Up	Lipids metabolism
LC	PC(16:0/16:0)	18131.71	16678.20	37543.99	9896.96	Down	Lipids metabolism
LC	PC(20:3(8Z,11Z,14Z)/20:1(11Z))	624.09	216.21	1035.73	453.21	Down	Lipids metabolism
LC	PC(20:4(8Z,11Z,14Z,17Z)/22:6(4Z,7Z,10Z,13Z,16Z,19Z))	366.25	347.74	742.80	329.84	Down	Lipids metabolism
LC	PC(MonoMe(9,5)/MonoMe(11,5))	1264.72	142.73	1446.07	110.74	Down	Lipids metabolism
LC	PC(o-22:0/20:1(11Z))	318.10	102.75	543.21	255.53	Down	Lipids metabolism
LC	PE(14:0/22:6(4Z,7Z,10Z,13Z,16Z,19Z))	403.12	403.02	795.90	431.94	Down	Lipids metabolism
LC	PE(18:1(11Z)/18:2(9Z,12Z))	191.14	137.36	399.15	186.38	Down	Lipids metabolism
LC	PE(20:4(8Z,11Z,14Z,17Z)/18:2(9Z,12Z))	368.20	354.11	771.95	340.10	Down	Lipids metabolism
LC	PE(20:4(8Z,11Z,14Z,17Z)/20:4(8Z,11Z,14Z,17Z))	1643.69	1269.10	2739.03	886.80	Down	Lipids metabolism
LC	PE(22:2(13Z,16Z)/18:1(11Z))	690.61	568.32	1272.60	556.66	Down	Lipids metabolism
LC	PE(22:4(7Z,10Z,13Z,16Z)/22:6(4Z,7Z,10Z,13Z,16Z,19Z))	1271.84	682.56	1858.62	449.82	Down	Lipids metabolism
LC	PE(22:4(7Z,10Z,13Z,16Z)/P-18:0)	755.52	157.46	1100.13	378.12	Down	Lipids metabolism
LC	PE(22:6(4Z,7Z,10Z,13Z,16Z,19Z)/18:0)	10704.76	8068.20	17228.92	3879.06	Down	Lipids metabolism
LC	PE(22:6(4Z,7Z,10Z,13Z,16Z,19Z)/18:1(9Z))	4427.50	3116.73	7734.83	3047.53	Down	Lipids metabolism
LC	PE(22:6(4Z,7Z,10Z,13Z,16Z,19Z)/20:4(5Z,8Z,11Z,14Z))	3642.89	839.27	4517.20	745.87	Down	Lipids metabolism
LC	PE(O-16:1(1Z)/22:6(4Z,7Z,10Z,13Z,16Z,19Z))	22646.23	15229.81	35307.36	9030.59	Down	Lipids metabolism

LC	PE(P-18:1(9Z)/22:6(4Z,7Z,10Z,13Z,16Z,19Z))	2209.94	1858.70	3678.05	1164.39	Down	Lipids metabolism
LC	PG(16:0/20:4(5Z,8Z,11Z,14Z))	956.96	933.93	2232.89	793.32	Down	Lipids metabolism
LC	PG(16:0/22:4(7Z,10Z,13Z,16Z))	756.05	456.13	1350.02	575.77	Down	Lipids metabolism
LC	PG(18:3(9Z,12Z,15Z)/18:0)	882.83	301.42	1281.93	206.02	Down	Lipids metabolism
LC	PG(18:3(9Z,12Z,15Z)/18:1(9Z))	1577.92	356.75	2066.93	231.96	Down	Lipids metabolism
LC	PGP(18:0/22:4(7Z,10Z,13Z,16Z))	282.29	248.21	591.52	194.05	Down	Lipids metabolism
LC	PGP(18:1(11Z)/22:5(7Z,10Z,13Z,16Z,19Z))	2731.28	2286.53	6988.24	3486.28	Down	Lipids metabolism
LC	PGP(18:2(9Z,12Z)/20:3(8Z,11Z,14Z))	241.07	234.26	524.34	227.00	Down	Lipids metabolism
LC	PI(20:4(8Z,11Z,14Z,17Z)/16:0)	1938.59	1400.40	3612.22	1050.28	Down	Lipids metabolism
LC	PI(22:5(4Z,7Z,10Z,13Z,16Z)/16:0)	211.75	199.34	459.53	181.32	Down	Lipids metabolism
LC	PI(22:5(7Z,10Z,13Z,16Z,19Z)/16:0)	1696.96	1542.73	3680.54	1444.63	Down	Lipids metabolism
LC	PS(18:0/16:1(9Z))	415.51	391.69	1123.24	654.00	Down	Lipids metabolism
LC	PS(18:0/22:6(4Z,7Z,10Z,13Z,16Z,19Z))	9351.44	4212.25	13096.06	3211.14	Down	Lipids metabolism
LC	PS(22:6(4Z,7Z,10Z,13Z,16Z,19Z)/18:0)	5204.97	3864.22	8748.21	3041.56	Down	Lipids metabolism
LC	SM(d18:0/12:0)	863.69	635.29	1458.72	493.17	Down	Lipids metabolism
LC	SM(d18:1/18:1(9Z))	1081.16	159.07	1364.70	329.20	Down	Lipids metabolism
LC	TG(18:4(6Z,9Z,12Z,15Z)/18:4(6Z,9Z,12Z,15Z)/18:4(6Z,9Z,12Z,15Z))	519.01	130.45	819.92	343.43	Down	Lipids metabolism
LC	TG(20:5(5Z,8Z,11Z,14Z,17Z)/18:4(6Z,9Z,12Z,15Z)/o-18:0)	363.09	88.13	568.98	235.42	Down	Lipids metabolism
GC	(S)-Mandelic acid	0.71	0.11	0.35	0.33	Up	Others
LC	15beta-Hydroxydesogestrel	1775.60	131.86	1552.35	92.13	Up	Others
LC	1-Arachidonoylglycerophosphoinositol	1117.48	365.21	1555.03	333.54	Down	Others
LC	1-Hydroxy-1-phenyl-3-hexadecanone	1824.88	494.99	2653.49	610.55	Down	Others
GC	1-Hydroxyanthraquinone	2.36	0.64	1.59	0.38	Up	Others

LC	2-Acetyl-3-methylthiophene	272.54	94.72	530.71	99.92	Down	Others
LC	2-Arachidonylglycerol	6839.65	276.96	6430.07	107.16	Up	Others
LC	2-Dodecylbenzenesulfonic acid	12382.74	515.55	11500.69	500.76	Up	Others
LC	2-Phenylaminoadenosine	810.62	161.37	992.53	33.56	Down	Others
GC	3-Aminoisobutyric acid	59.94	13.01	31.44	10.71	Up	Others
LC	3-Hydroxyisovaleryl-CoA	366.47	102.42	603.68	264.37	Down	Others
LC	3-O-Sulfogalactosylceramide (d18:1/22:0)	381.81	344.03	692.59	274.29	Down	Others
LC	4,8,12,15,19-Docosapentaenoic acid	683.93	213.35	903.75	244.40	Down	Others
GC	4-aminobutyric acid	26.41	10.70	9.89	2.37	Up	Others
GC	4-Hydroxymandelic acid	0.39	0.09	0.28	0.07	Up	Others
LC	5a-Androstane-3b,17b-diol	1818.63	1508.88	483.99	132.16	Up	Others
LC	8-Oxo-dGMP	905.06	188.96	1078.56	170.79	Down	Others
LC	Acetyl-CoA	422.34	133.60	672.59	283.34	Down	Others
GC	Allo-inositol	0.20	0.05	0.10	0.09	Up	Others
GC	alpha-Aminoadipic acid	0.06	0.05	0.12	0.03	Down	Others
LC	Alpha-CEHC	2772.15	197.97	2526.91	98.58	Up	Others
LC	alpha-Hydroxytamoxifen	320.51	252.72	78.04	25.89	Up	Others
LC	Alpha-Linolenic acid	357.67	333.94	65.13	18.04	Up	Others
GC	Aminomalonic acid	0.54	0.15	0.86	0.17	Down	Others
LC	CE(15:0)	949.16	146.00	1167.43	124.94	Down	Others
LC	Cer(d18:1/18:1(11Z))	1846.96	1420.83	3398.31	1431.29	Down	Others
LC	Citalopram propionic acid	805.16	215.84	1017.04	33.47	Down	Others
GC	creatine degr	1.80	1.79	0.18	0.15	Up	Others
LC	Cysteineglutathione disulfide	1737.78	616.87	2833.23	962.02	Down	Others
LC	Docosahexaenoic acid	11552.43	2029.52	13670.55	2060.71	Down	Others

LC	Ethyl tetradecanoate	2607.59	332.01	3343.01	805.81	Down	Others
GC	Hesperitin	5.77	1.03	4.41	0.82	Up	Others
LC	Hydrogen phosphate	5352.26	251.43	5101.68	156.61	Up	Others
LC	L-Cysteinylglycine disulfide	354.09	123.59	578.45	188.17	Down	Others
GC	malonic acid	0.66	0.18	0.48	0.05	Up	Others
GC	Methyl Palmitoleate	0.38	0.10	0.29	0.04	Up	Others
GC	Methylmalonic acid	0.58	0.82	1.93	0.30	Down	Others
GC	nicotinamide	3.89	0.21	4.12	0.17	Down	Others
LC	N-Undecylbenzenesulfonic acid	7714.09	320.14	7173.04	310.91	Up	Others
GC	O-methylthreonine	0.26	0.11	0.13	0.16	Up	Others
GC	O-Phosphorylethanolamine	2.02	0.43	3.60	0.85	Down	Others
GC	phosphate	19.30	2.07	16.08	1.68	Up	Others
GC	phosphomycin	13.74	1.76	11.53	1.17	Up	Others
LC	Pipericine	1549.97	346.89	1265.61	98.38	Up	Others
LC	Piperochromenoic acid	2483.42	89.96	2312.74	74.94	Up	Others
LC	Ribose-1-arsenate	886.94	265.16	682.78	110.47	Up	Others
LC	S-3-oxodecanoyl cysteamine	1248.39	773.74	635.98	128.30	Up	Others
GC	stearic acid	16.63	3.80	13.67	1.58	Up	Others
GC	terephthalic acid	0.33	0.11	0.22	0.05	Up	Others
LC	Zinc acetate	844.14	287.77	585.49	217.85	Up	Others
